# Population-level coding of avoidance learning in medial prefrontal cortex

**DOI:** 10.1038/s41593-024-01704-5

**Published:** 2024-07-29

**Authors:** Benjamin Ehret, Roman Boehringer, Elizabeth A. Amadei, Maria R. Cervera, Christian Henning, Aniruddh R. Galgali, Valerio Mante, Benjamin F. Grewe

**Affiliations:** 1 Institute of Neuroinformatics, University of Zurich and ETH Zurich, Zurich, Switzerland; 2 Gatsby Computational Neuroscience Unit, University College London, London, UK; 3 ETH AI Center, ETH Zurich, Zurich, Switzerland; 4 University Research Priority Program (URPP) Adaptive Brain Circuits in Development and Learning (AdaBD), University of Zurich, Zurich, Switzerland

**Keywords:** Learning and memory, Computational neuroscience, Sensorimotor processing

## Abstract

The medial prefrontal cortex (mPFC) has been proposed to link sensory inputs and behavioral outputs to mediate the execution of learned behaviors. However, how such a link is implemented has remained unclear. To measure prefrontal neural correlates of sensory stimuli and learned behaviors, we performed population calcium imaging during a new tone-signaled active avoidance paradigm in mice. We developed an analysis approach based on dimensionality reduction and decoding that allowed us to identify interpretable task-related population activity patterns. While a large fraction of tone-evoked activity was not informative about behavior execution, we identified an activity pattern that was predictive of tone-induced avoidance actions and did not occur for spontaneous actions with similar motion kinematics. Moreover, this avoidance-specific activity differed between distinct avoidance actions learned in two consecutive tasks. Overall, our results are consistent with a model in which mPFC contributes to the selection of goal-directed actions by transforming sensory inputs into specific behavioral outputs through distributed population-level computations.

## Abstract (web summary)

Ehret et al. uncover neural activity patterns in the prefrontal cortex that link sensory stimuli to learned behavioral responses by isolating interpretable activity patterns that are shared among mice performing the same task.

## Main

Learning to appropriately respond to sensory information that is predictive of threats or rewards is a vital skill for every animal. This learning process depends on a network of interconnected brain regions involved in diverse functions such as sensory processing, the learning of stimulus-outcome associations and behavioral execution. In rodents, the medial prefrontal cortex (mPFC) has been implicated in linking sensory information to appropriate actions during learning and behavior execution in various forms of conditioning^1^. Specifically, mPFC neurons acquire strong and temporally precise responses to behaviorally relevant stimuli over learning^1–3^. Moreover, optogenetic manipulations of prefrontal activity can drive and/or inhibit behavioral execution in a variety of paradigms, such as fear conditioning^4–6^, active avoidance^7,8^, reward-based conditioning^3,9^ and conditioned place preference^10^. Additionally, mPFC has a crucial role in the selection between different response options^11^ and in switching between different learned stimulus-response associations^12–14^.

While it is well established that behaviorally relevant sensory stimuli can elicit mPFC activity and that such activity can influence behavior, it is still unclear (1) how sensory-evoked mPFC activity is locally organized and transformed to drive specific actions and (2) how such transformations are updated to enable behavioral flexibility. Investigating these questions has been challenging due to the properties of mPFC neural activity and the limitations of traditional experimental strategies and analysis approaches. First, learned, action-related activity is hard to distinguish from the pronounced general motion-related activity found in mPFC^15–17^. Second, stimuli and behavioral responses often show a temporal overlap inherent to task design, complicating the isolation of sensory- and behavior-related neural activity. Third, prefrontal neurons might show mixed selectivity for multiple task variables^18^. Finally, due to this temporal and spatial mixing, optogenetic approaches have limited ability to manipulate specific task-related signals as these do not necessarily align with cell types or projection-specific subpopulations that could be targeted selectively.

Here we addressed these issues by performing large-scale neuronal recordings during a mouse active avoidance paradigm with changing contingencies between stimulus and conditioned responses. This experimental approach, combined with a new data analysis pipeline, allowed us to isolate neural correlates of individual task variables and to study changes in the neural correlates of stimuli and behaviors throughout learning.

## Results

### A new active avoidance paradigm allows linking a sensory stimulus to two different behavioral responses

**Fig. 1:**
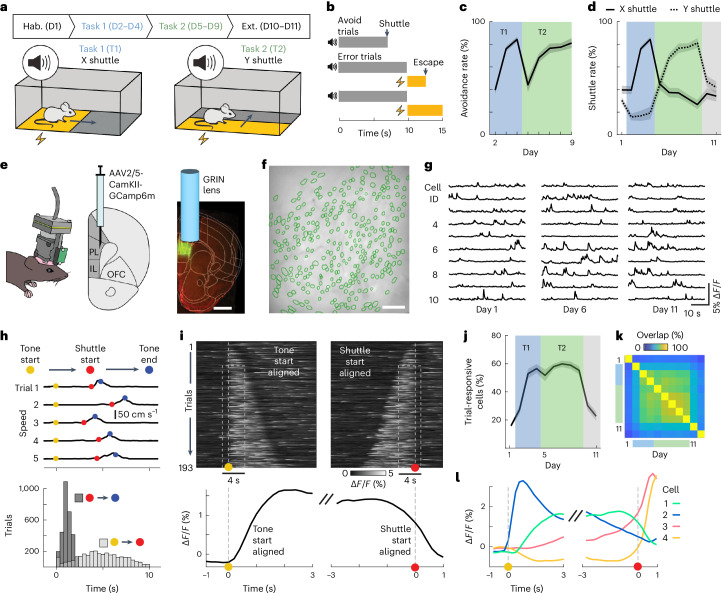
The two-dimensional active avoidance paradigm and recording of prefrontal population activity. **a**, Task schematic and time course of the 11-day learning paradigm. Tasks 1 and 2 are defined by shuttling along the *x* and *y* axes of the shuttle box, respectively. **b**, Trial structure and illustration of the different trial types (avoid and error). **c**, Percentage of successful avoid trials per active avoidance session (*n* = 12 mice, mean ± s.e.m.). **d**, Shuttle rates for X shuttle (solid line) and Y shuttle (dashed line) across 11 days of learning (*n* = 12 mice, mean ± s.e.m.). **e**, Miniaturized (single photon) population calcium imaging in freely behaving mice. GCaMP6m was genetically expressed in pyramidal neurons, and a GRIN lens was implanted above the PL. Scale bar: 1 mm. **f**, Cell map of an example animal. Scale bar: 100 μm. **g**, Calcium fluorescence traces of ten example neurons on days 1, 6 and 11. **h**, Top, mouse speed for five exemplary avoid trials including markers for three reference time points (tone start, shuttle start and tone end). Bottom, distributions of latencies from tone start to shuttle start and shuttle start to tone end over all avoid trials (days 2–9, 12 mice). **i**, Top, calcium fluorescence traces of one example neuron aligned to tone start (left) or shuttle start (right). Trials are sorted according to trial length. Bottom, trial-averaged neuronal activity of the same neuron. **j**, Percentage of trial-responsive neurons across 11 days of learning (*n* = 12 mice, mean ± s.e.m.). See Methods for the definition of trial-responsiveness. **k**, Overlap of trial-responsive subpopulations across 11 days, where the overlap between days ***i*** and ***j*** is defined as *n*_*i* and *j*_/((*n*_*i*_ + *n*_*j*_)/2). **l**, Trial-averaged response of four example neurons aligned to tone start (left) or shuttle start (right). OFC, orbitofrontal cortex; IL, infralimbic cortex; PL, prelimbic cortex; D1, day 1; D2–D4, days 2–4; D5–D9, days 5–9; D10–D11, days 10–11.

**Extended Data Fig. 1:**
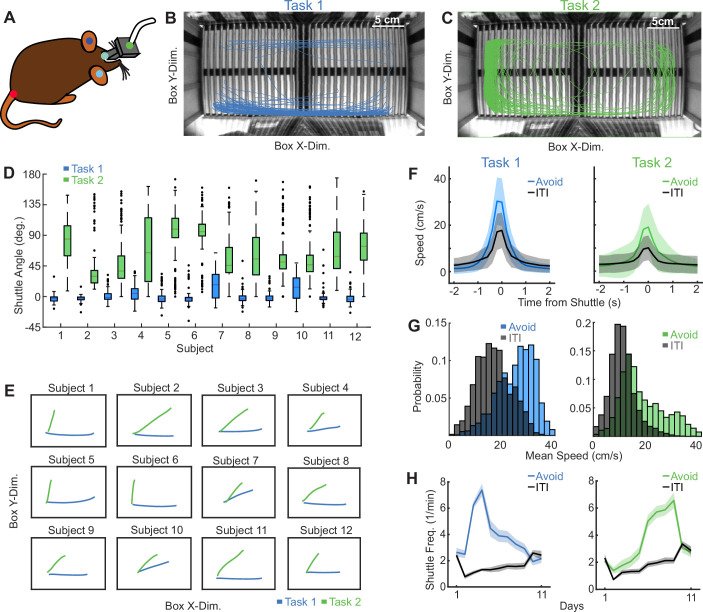
Animal tracking and behavior analysis. (**a**) DeepLabCut tracking points of the animal. (**b**,**c**) Avoidance trajectories during task 1 (X-shuttling) and task 2 (Y-shuttling), respectively. (**d**) Distribution of shuttle angles in degrees for all 12 animal subjects in tasks 1 and 2. Box plots indicate median (center), 25th and 75th percentiles (box) and most extreme data points (whiskers) that were not considered outliers (points for which the distance from the box exceeds 1.5 times the length of the box). (**e**) Mean shuttle trajectories depicted in the shuttle box for all 12 animals. (**f**) Animal speed time courses aligned to the shuttle start for tasks 1 (blue, left) and 2 (green, right) (mean ± s.d. over shuttles from 12 mice). ITI shuttles are depicted in gray. (**g**) Distributions of mean shuttle speed (average over 2 s window centered around shuttle) for ITI (gray) and avoid (colored) shuttles in tasks 1 (left, 1151 avoid shuttles, 1329 ITI shuttles) and 2 (right, 1945 avoid shuttles, 3859 ITI shuttles). (**h**) X axis (blue) and Y axis (green) shuttle frequency compared to the respective ITI (gray) shuttle frequency across days (mean ± s.e.m. over 12 mice).

We first developed a new 11-day instrumental conditioning paradigm for mice that we refer to as two-dimensional active avoidance. The paradigm consisted of habituation (day 1), active avoidance training (days 2–9) and extinction (days 10–11; Fig. 1a). Each session comprised 50 trials, each starting with the presentation of a tone (maximum duration: 10 s, 80 dB, 8 kHz). In active avoidance sessions, the tone was followed by an aversive foot shock (maximum duration: 5 s, 0.2 mA). On each trial, we defined a safe zone that covered half of the chamber and whose location depended on the position of the mouse at the trial start and the task type (see below). Mice could avoid the shock by moving into the safe zone during the tone presentation, which immediately terminated the trial (Fig. 1b). On days 2–4, mice were required to shuttle along the *x* axis of the box to reach the safe zone (Fig. 1a (task 1) and Supplementary Video 1). To study whether and how subjects could flexibly adapt their avoidance behavior, days 5–9 required shuttling along the perpendicular *y* axis (Fig. 1a (task 2) and Supplementary Video 1). If mice did not shuttle into the safe zone during the tone, the shock was delivered and two of the four movable platforms were elevated to mark the safe zone and to allow the animals to escape the shock by jumping on the platform (Methods). Trials in habituation and extinction sessions (days 1, 10 and 11) included tone presentations, but no shock presentations. For each trial in these sessions, the definition of the safe zone was randomly chosen to follow the logic of either task 1 or 2. As for the learning sessions, the tone was shut off or platforms were raised depending on the animal’s behavior. In the following text, we refer to trials that were terminated by the execution of the correct shuttle action during the tone as avoid trials and to trials that included a shock presentation as error trials (Fig. 1b). During task 1, the proportion of avoid trials increased from 40 ± 4% to 84 ± 2% (mean ± s.e.m.; Fig. 1c). After the task switch on day 5, performance dropped to 44 ± 6% but recovered to 81 ± 4% by the end of task 2. This recovery was based on mice adjusting their shuttle behavior toward the correct direction (Fig. 1d and Extended Data Fig. 1). While the Y-shuttling rate increased from 19 ± 6% to 81 ± 4% between days 4 and 9, X-shuttling concurrently dropped from 84 ± 2% to 27 ± 4%.

**Extended Data Fig. 2:**
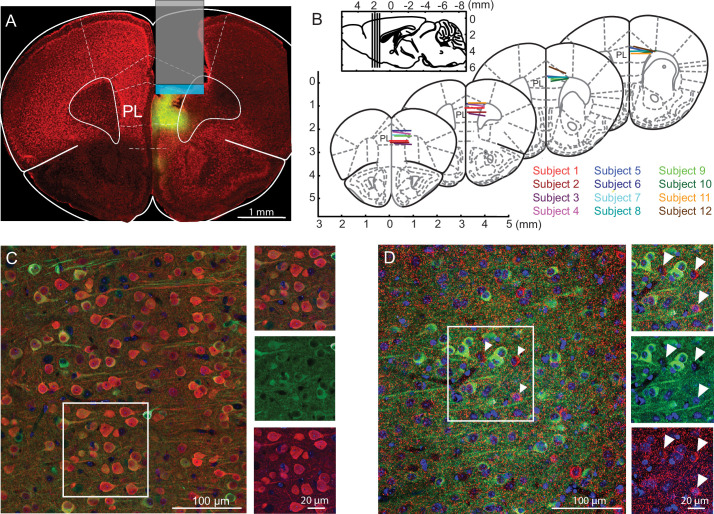
GRIN lens implant and cell labeling verification. (**a**) Verification of the placement of the GRIN lens, with the GRIN lens position displayed in an example coronal mouse brain section. Nissl stain (red) and GCaMP6m (green). The shade blue area below the GRIN lens corresponds to an estimate of the distance between the edge of the implant (displayed in **b**) and the imaging plane (200 μm). (**b**) Locations of the edge of the implant were mapped onto the mouse brain atlas (see ref. ^44^) for each individual animal. (**c**) Left: immunohistological validation of GCaMP6m expression in PL, comparing GCaMP6m (green) and neurogranin labeling (red) in excitatory neurons. Top right: overlap. Middle right: GCaMP6m. Bottom right: neurogranin. (**d**) Left: comparison of GCaMP6m expression (green) to GAD65 staining (red). Top right: overlap. Middle right: GCaMP6m-expressing neurons. Bottom right: GAD65-positive neurons. Arrows indicate three examples of GAD65-positive neurons. Five independent repetitions with images from different subjects produced qualitatively similar results (**c**,**d**). Source data

**Extended Data Fig. 3:**
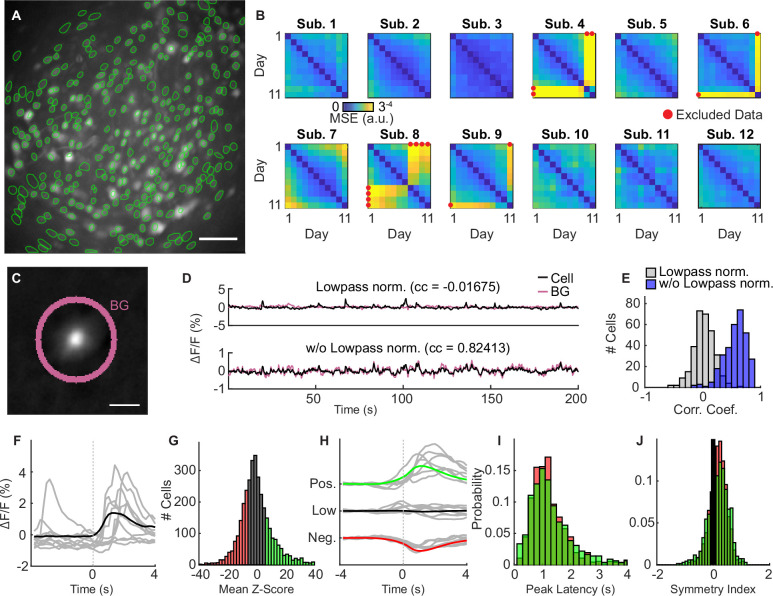
Validation of imaging and signal extraction techniques. (**a**) Map of identified cells (in green) overlaid on an imaging frame displaying the log of the standard deviation of individual pixels over all preprocessed, aligned and concatenated recording sessions for an example mouse. This visualization provides an intuition for the spatial extent of individual cells, but by itself cannot capture the quality of individual cells (see Extended Data Fig. 4). Scale bar: 100 μm. Five independent repetitions with data from different subjects produced qualitatively similar results. (**b**) Quantification of session alignment for individual subjects. We calculated pairwise mean squared errors (MSEs) between the cell maps of two aligned sessions. Shifts in the field of view that could not be aligned were visible as high MSE values between a given session and a set of well-aligned sessions. Data exclusions are displayed in red. (**c**) Analysis of background (BG) activity and the effect of lowpass normalization. For all cells of an example session (example cell displayed in grayscale), we calculated background activity traces using ring filters centered on the cell’s centroid (displayed in purple). Scale bar: 30 μm. (**d**) Activity traces for the two spatial filters from **c** with and without lowpass normalization. Without normalization, cell and background activities are highly correlated, indicating contamination of the cellular signal. With normalization, the correlation disappears and calcium transients can be resolved in the cellular signal. (**e**) Quantification of the correlation between cellular and background activity for all cells of an example session. Without lowpass normalization, most cells showed substantial positive correlations (median Pearson correlation coefficient = 0.60). Using the lowpass normalization, the median Pearson correlation coefficient dropped to 0.02, indicating that the lowpass filtering strategy employed in our pipeline successfully removes the majority of neuropil contamination from the cells’ activities. (**f**) Analysis of motion-related activity as a control for motion-related artifacts in neural activity. Average response to all ITI shuttles (black) of an example cell with individual example shuttles displayed in gray. This example cell shows motion-related activity based on calcium transients that are not consistent with artifacts based on microscope motion. (**g**) Quantification of motion-related responses over all recorded cells. Motion score is calculated as the mean z score in the 4 s after motion onset (at time 0). Many cells show positive (green) and negative (red) responses, but many cells (gray) are not strongly modulated by motion, indicating that there is no systematic motion artifact affecting all cells. (**h**) The time course of positively and negatively modulated cells is consistent with neural responses (slow and asymmetric) rather than motion-related artifacts, which would be expected to be fast and symmetric. (**i**) Quantification of peak latency for positively and negatively modulated cells. (**j**) Quantification of response symmetry for positively and negatively modulated cells. Symmetry index is calculated as (activity_peak + 2 s_ + activity _peak − 2 s_)/activity_peak_, such that 0 indicates symmetric responses.

**Extended Data Fig. 4:**
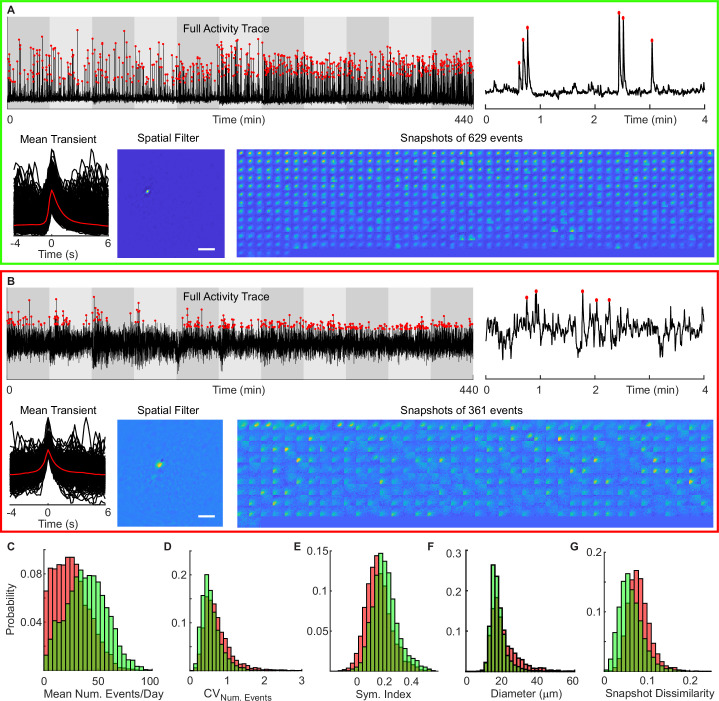
Quality control for identified neurons. (**a**) Features of an example cell that was accepted in the annotation process. A cell is defined by its activity trace over all sessions (top left, individual imaging sessions are indicated as different gray shades, see top right for zoom-in) and its spatial filter (bottom middle). Scale bar: 100 μm. We detect events (red dots) as peaks in the activity trace that deviate 3 standard deviations from the mean. We then use these detected events to calculate a mean transient (bottom left in red, individual events in black) and to display snapshots of the images that caused the peaks in the activity trace (bottom right). Cells are accepted if they have (1) a clear and appropriately shaped spatial extent, (2) a stable activity trace with well-identified peaks, (3) a mean transient with fast rise and slow decay as expected from calcium indicator kinematics and (4) if the event snapshots consistently resemble the spatial filter (displayed on the upper left of the snapshot matrix). Snapshots that do not resemble the filter indicate contamination through another cell. (**b**) Example of a rejected cell with a noisy spatial filter and activity trace, a symmetric mean transient and inconsistent event snapshots. (**c**) Distributions of mean numbers of events per day. (**d**) Distributions for the coefficient of variation of the number of events per day (ratio of s.d. and mean). The CV tends to be substantially below 1 (median = 0.52 for accepted cells), indicating that the distribution of the number of events per day does not fluctuate far from the mean over days (displayed in **c**), which suggests stable activity levels over sessions. (**e**) Distributions of symmetry index calculated using the mean transient as (activity_peak + 1 s_ + activity_peak − 1 s_)/activity_peak_. (**f**) Distribution of filter diameter for accepted (green) and rejected cells (red). (**g**) Distributions of snapshot dissimilarity values. Snapshot dissimilarity is calculated as the mean of the mean squared error (MSE) between the filter and each event snapshot.

To investigate the neural correlates of the learned avoidance behaviors in mPFC, we expressed the genetically encoded calcium indicator GCaMP6m in excitatory neurons of the prelimbic area (Fig. 1e and Extended Data Fig. 2) and used miniaturized fluorescence microscopy to image population activity in freely behaving mice (Supplementary Video 2). This allowed us to record and track the activity of 3,333 mPFC excitatory neurons in 12 mice (278 ± 50 neurons, mean ± s.d. over mice) throughout the whole 11-day paradigm (Fig. 1f,g and Extended Data Fig. 3 and 4).

**Extended Data Fig. 5:**
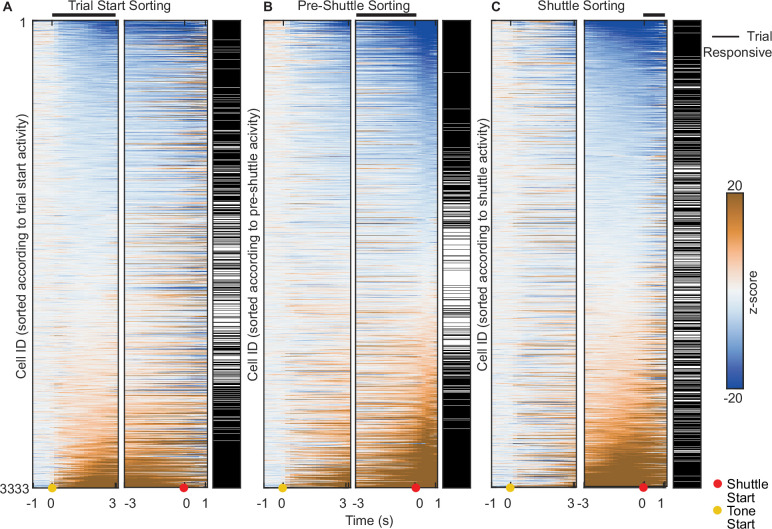
Diversity of single-cell response profiles. (**a**) Trial-averaged and z-scored neural activity (Methods) of all recorded cells during avoid trials of an example session (session 7). Activity is aligned to tone start (left) or shuttle start (right), as shown, for example, cells, in Fig. 1l. Cells are sorted according to their time-averaged z score in the 3 s window after tone start (window is indicated by the black bar at top of panel). This sorting reveals that cells tend to have different responses in the tone start and shuttle start windows. Barcode indicates if cells were classified as trial-responsive (black) or not (white). (**b**) Same as **a**, but cells are sorted according to their mean *z* score in the 3 s window preceding shuttle start. This sorting reveals that the activity preceding shuttle actions tends to be different from both the activity at tone start and the activity after shuttle start. (**c**) Same as **a**, but cells are sorted according to their mean z score in the 1 s window after shuttle start. Overall, responses are highly diverse over the population because cells show different combinations of activity levels for the different task-related events.

To analyze the recorded neural activity during avoid trials, we first aligned recordings to the following two key events within each trial to account for trial-to-trial variability: tone start and shuttle start (Fig. 1h). In a window around these alignment time points, sensory stimulation and behavior were consistent over trials, such that we could compute trial averages and jointly analyze neural responses from multiple trials (Fig. 1i). We found that during active avoidance sessions, 54 ± 3% (mean ± s.e.m., *n* = 12 mice) of all recorded cells showed significantly different activity during the trial window (tone start to shuttle start) as compared to baseline periods (Fig. 1j). This fraction was substantially lower in habituation (15 ± 1%) and extinction sessions (26 ± 3%), and the overlap between the classified cell subsets was high between avoidance sessions (60 ± 2%), but low between extinction sessions (28 ± 5%; Fig. 1k). These results suggest that mPFC is recruited for sensory processing and/or production of avoidance behavior during active avoidance sessions. The responses of individual cells were highly diverse (Fig. 1l and Extended Data Fig. 5). While some cells’ activity clearly aligned with the tone or the avoidance action, other cells showed diverse temporal dynamics. Because it was difficult to isolate neuronal signals specific to the sensory stimulus, motion and avoidance action on the single-cell level, we next turned to population-level decoding approaches.

### Alignment of neural recordings from different mice into a joint subspace

**Fig. 2:**
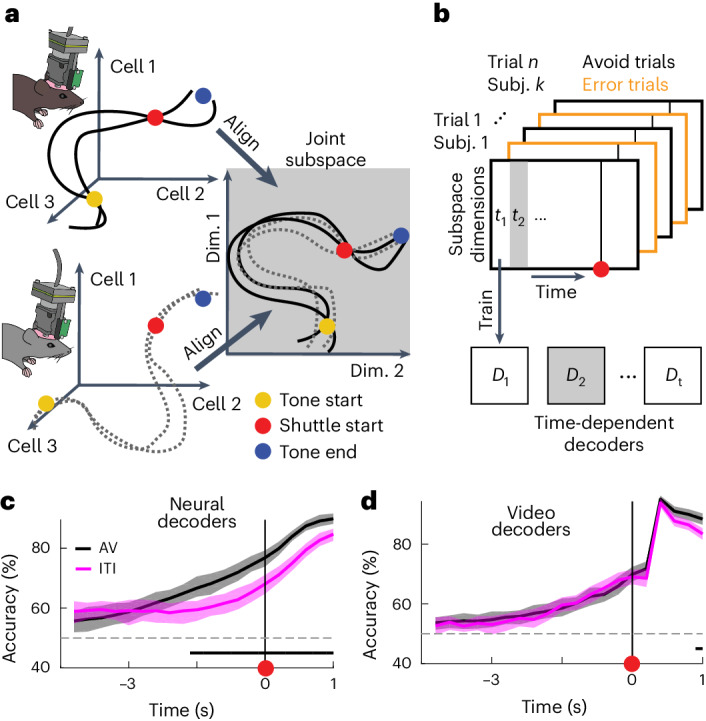
Subject alignment and prediction of avoidance actions. **a**, Illustration of the neuronal subspace alignment procedure across animals (see Extended Data Fig. 6 for details). **b**, Schematic representation of the decoding approach to predict avoidance behavior from mPFC neuronal activity. For each time step (*t*_1_, *t*_2_, etc.), an individual decoder (*D*_1_ to *D*_t_) was trained to predict the trial outcome (avoid or error). **c**, Decoding accuracies across time for decoding of avoid versus error trials (AV, black) and ITI shuttles versus random ITI periods (ITI, magenta; mean and 95% CIs for 80 repetitions of the analysis using different samples of trials; Methods). Black bar indicates significant differences between the AV and ITI settings based on nonoverlapping CIs. **d**, Same as **c**, but for decoders trained using the animals’ speed extracted from video tracking data.

**Extended Data Fig. 6:**
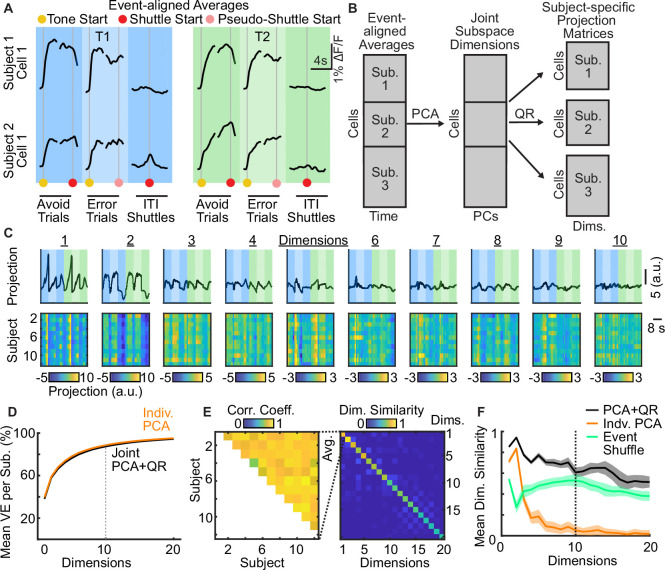
Alignment of neural data across animals into a joint subspace. (**a**) Specification of time points used for alignment displayed for two example neurons from different subjects that show similar responses during avoid, error and ITI events in task 1 (blue shade) and task 2 (green shade). (**b**) To align neural population data, the temporally aligned event averages displayed in **a** are first concatenated for all cells. We then process these event averages (Methods) and concatenate them along the time axis. Next, PCA is used to generate the joint subspace, which is defined by the coefficients of the first n PCs (n is chosen below). Subject-specific projection matrices into the joint subspace can then be computed by splitting the coefficient matrix back into matrices for individual subjects and orthogonalizing them using the QR decomposition. (**c**) Top: mean projections onto subspace dimensions 1–10 (n = 12 subjects). Shading indicates temporal structure displayed in **a**. Bottom: projections displayed for individual subjects, highlighting common structure. (**d**) Variance explained by the first 20 subspace dimensions (mean and 95% CIs for 80 repetitions) for the joint PCA + QR procedure (black). To control how the alignment procedure affects how well the low-dimensional subspace captures neural variability, we performed PCA individually per subject as an upper baseline for the explained variance (orange). The alignment only has a minor effect on the variance explained by the identified subspace. In this work, we use the first 10 dimensions to define the joint subspace. (**e**) Left: cross-subject correlation of the first subspace dimension calculated for pairs of projections into this dimension (see bottom row of **c**). Right: similarity of pairs of dimensions, where similarity is computed by averaging the elements of the triangular cross-subject correlation matrix displayed on the left. (**f**) Average dimension similarity for the first 20 subspace dimensions with alignment (PCA + QR, black) and without alignment (Indv. PCA, orange) (mean and 95% CIs for 80 repetitions). Additionally, we controlled how the dimension alignment quality depends on the temporal alignment of specific activity patterns around the chosen events (rather than general bump-like activity) by shuffling event types between subjects (event shuffle, green). We found that shuffling events led to a marked drop in alignment quality, indicating that the correct alignment of neural subspaces depends on the correct temporal alignment of conceptually similar events. We chose the number n of used dimensions to be 10, as it constitutes a good tradeoff between explained variance (see **c**) and alignment quality.

Decoding approaches allowed for identifying and capturing differences in neural population activity between trial types (for example, avoidance versus error trials). Generally, such approaches are well suited in settings where the number of samples (here trials) exceeds the number of dimensions (here cells). In typical neuroscience settings, however, we record high-dimensional neural signals (many cells), but only have a few behavioral trials per subject. To facilitate decoding analyses, we asked if we could jointly analyze trials of different subjects in a low-dimensional coding subspace that is aligned between subjects (Fig. 2a). This approach required the recorded neural activity to have the following two properties: (1) the high-dimensional recordings can be well described by low-dimensional trajectories in the state-space spanned by the recorded cells and (2) the task-related neural activity follows similar dynamics over subjects. Using a dimensionality reduction and alignment procedure (details in Methods), we confirmed that our data satisfy these two properties (Extended Data Fig. 6). We calculated task-related neural activity for all cells as event-aligned activity averages for avoid trials, error trials and shuttles in the intertrial interval (ITI; Extended Data Fig. 6a). We first showed that for individual subjects, more than 90% of the neural variability could be explained by less than 15 dimensions (Extended Data Fig. 6d (orange line)). Next, we showed that by aligning the state-spaces of individual subjects we could define one single joint subspace that shows only slight decreases in explained variance in comparison to the subject-specific subspaces (Extended Data Fig. 6d (black line)). The fact that a single joint subspace can capture variability for all subjects shows that task-related neural dynamics are highly similar between subjects. Finally, we quantified the alignment quality for the individual dimensions of the joint subspace and found that a ten-dimensional subspace constitutes a good tradeoff between alignment quality and a fraction of explained variance (Extended Data Fig. 6e,f). In the following, we thus jointly analyze neural data from all subjects and perform decoding analyses in the ten-dimensional subspace.

### Avoidance-related activity is distinct from activity related to general motion

To test if mPFC population activity contained predictive information about upcoming avoidance actions, we trained decoders to discriminate neural activity data from avoid and error trials projected into the joint subspace (Fig. 2b). To capture dynamical processes during the trial, we trained individual support vector machine (SVM) decoders for every time step on temporally aligned trials. We aligned the avoid trials using the shuttle start as the alignment point. For error trials, however, this alignment point does not exist. We thus sampled an alignment point for each error trial (pseudoshuttle start), such that the distribution of trial lengths (Fig. 1h) matched the one of avoid trials. This prevented the trial length from being informative about the trial type.

**Extended Data Fig. 7:**
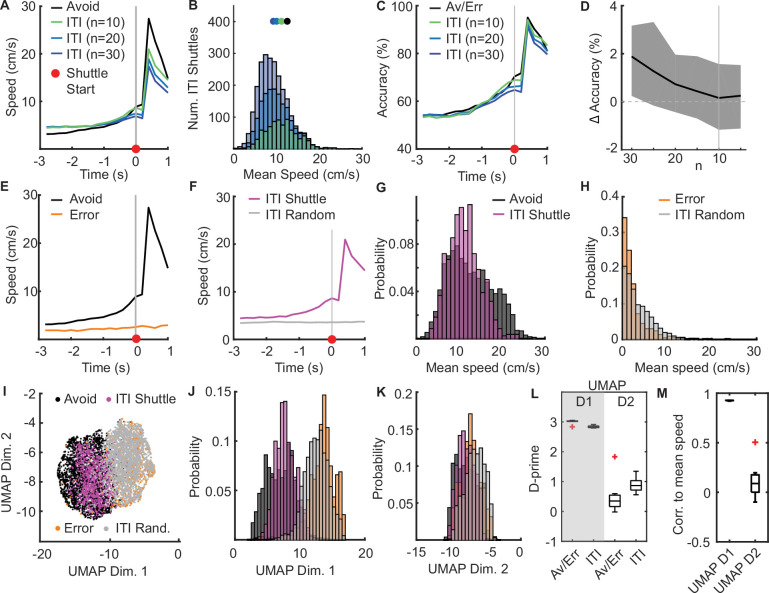
Selection of ITI shuttles and comparison to avoid shuttles. (**a**) Mean speed of ITI and avoid shuttles for different values of n, where n refers to the n fastest shuttles selected from each session. With decreasing n, the average speed trace of ITI shuttles becomes more similar to the one of avoid shuttles. (**b**) Distribution of mean speed (average over 2 s window centered around shuttle start) values of ITI shuttles for different values of n. Dots indicate distribution mean, which shifts toward that of avoid shuttles (black). (**c**) Decoding accuracy of video decoders trained to discriminate avoid and error trials (black) or ITI shuttles vs. random ITI periods. (**d**) Difference in predictive accuracy in **c** (average accuracy 2 s before shuttle start) between avoid/error and ITI decoding for different values of n (mean and 95% CIs for 80 repetitions). (**e**,**f**) Mean speed for the avoid and error (**e**) and ITI shuttle and ITI random (**f**) comparisons for n = 10. While the exact speed of avoid and ITI shuttles is not matched, the difference to error trials/random ITI periods is comparable. (**g**,**h**) Distributions of mean speed for avoid and ITI shuttles (**g**) and error trials and random ITI periods (**h**). While differences between the settings remain, the distributions of mean speed largely overlap. (**i**–**k**) UMAP embeddings of the 100-dimensional motion traces (5 tracking points × 20 time steps). Dimension 1 separates shuttles from non-shuttles. Avoid and ITI shuttles as well as error trials and random ITI periods are intermingled. (**l**) Discriminability index for the avoid and ITI settings quantified for the two UMAP dimensions (*n* = 10 random initializations). Dimension 1 consistently distinguishes between shuttles and non-shuttles in both settings, while dimension 2 does not. (**m**) Correlation of UMAP dimensions and mean speed (n = 10 random initializations). High correlation for dimension 1 indicates that mean speed is the main feature that distinguishes the different motion traces. Box plots indicate median (center), 25th and 75th percentiles (box) and most extreme data points (whiskers) that were not considered outliers (points for which the distance from the box exceeds 1.5 times the length of the box).

Consistent with previous work^8^, we found that decoding accuracy increased toward the shuttle action and was above chance levels before shuttle start (Fig. 2c), indicating that mPFC population activity contained predictive information about avoidance actions. To test if this effect was specific to avoidance actions or was rather a general property of the shuttle motion, we trained an additional set of SVM decoders to discriminate between spontaneous shuttles in the ITI versus randomly sampled ITI periods (Fig. 2c). To ensure that accuracy differences between avoid shuttle and ITI shuttle decoding could not be explained by differences in motion kinematics, we chose ITI shuttles such that the predictive information contained in the associated motion tracking data was comparable to avoid shuttles (Extended Data Fig. 7). We quantified this predictive information by training a set of decoders using video tracking data (Fig. 2d), which showed no difference between the AV (avoid) and ITI settings (as intended by the procedure detailed in Extended Data Fig. 7). In contrast, for neural decoders (Fig. 2c), ITI decoding accuracies were lower than for the avoid versus error setting, although they also exceeded chance levels. Together, these findings show that mPFC activity encodes information about upcoming avoidance actions, which cannot solely be explained by correlates of general motion. However, it remains unclear how the neural correlates of avoidance and motion relate to each other. We thus next assessed whether we could disentangle these signals during avoidance trials.

**Fig. 3:**
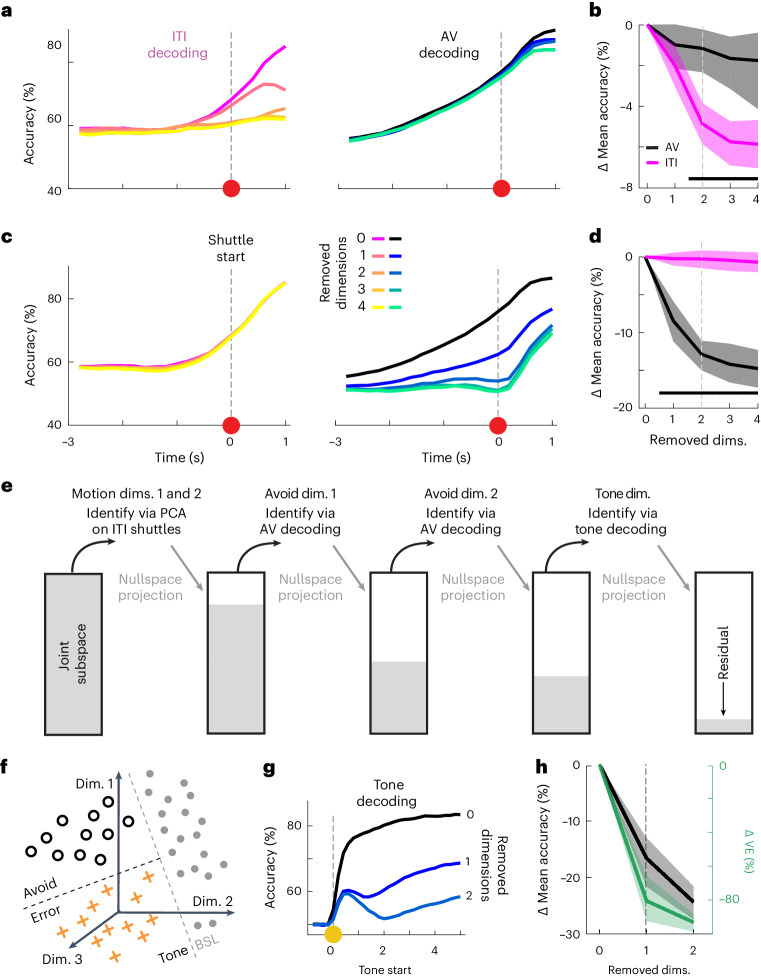
Decomposition of mPFC population activity into dimensions related to motion, avoidance actions and tone stimuli. **a**, Mean accuracy of neural decoders for ITI (left) or avoid shuttles (right) after the progressive removal of up to four motion dimensions (*n* = 80 repetitions). **b**, Drop in time-averaged accuracy (−3 s to 1 s) of ITI and avoid decoders from **a** with respect to the baseline setting (0 dimensions removed). **c**, Mean accuracy of neural decoders for ITI (left) or avoid shuttles (right) after the progressive removal of up to four avoid dimensions (*n* = 80 repetitions). **d**, Drop in time-averaged accuracy (−3 s to 1 s) of ITI and avoid decoders from **c**. **e**, Schematic representation showing the progressive decomposition of the joint subspace into five coding dimensions and a residual space. **f**, Schematic representation illustrating tone versus BSL decoding. **g**, Tone decoding accuracies after progressive removal of up to two-tone dimensions. **h**, Drop in time-averaged tone decoding accuracy (0–4 s) of the decoders from **g** and drop in VE for the respective decoding dimensions. In **b**, **d** and **h**, lines and shaded areas correspond to mean and 95% CIs for 80 repetitions, and vertical dotted lines correspond to the number of dimensions chosen for the subspace decomposition. Black bars in **b** and **d** indicate significant differences between the AV and ITI settings based on nonoverlapping CIs. BSL, baseline.

**Extended Data Fig. 8:**
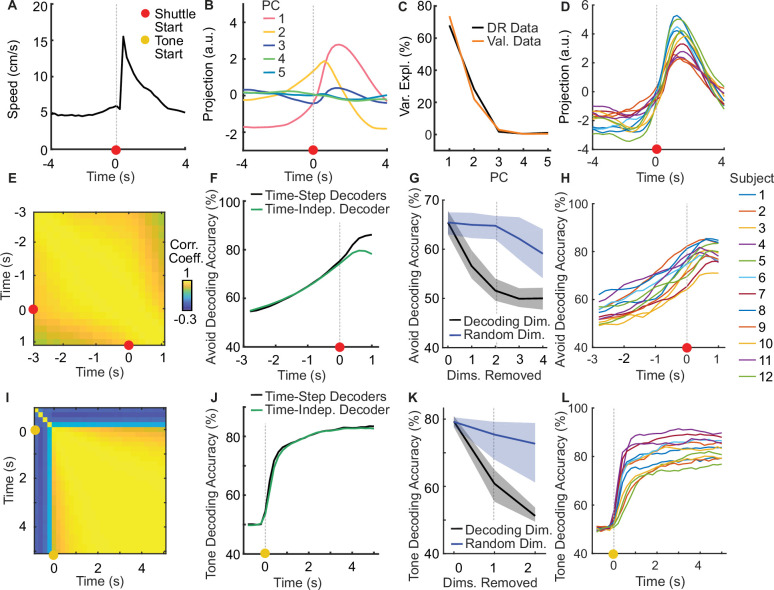
Definition of coding dimensions. (**a**) Mean animal speed during ITI shuttles used for the definition of motion dimensions (n = 1568 ITI shuttles). (**b**) To define motion dimensions, we computed the average neural activity in the joint subspace over all ITI shuttles and performed PCA. (**c**) Variance is explained by the first five PCs evaluated for the ITI shuttles used in dimensionality reduction (DR data) and for the ITI shuttles used in decoding analyses (val. data). Mean and 95% CIs for 80 repetitions. (**d**) PC1 projections for ITI shuttles of individual subjects. While there are differences in the magnitude, the temporal evolution of the projections is highly similar to the one of PC1 obtained using the pooled data (red line in **b**, Pearson correlation coefficient 0.983 ± 0.010, mean ± s.d. over 12 subjects). (**e**) Correlation coefficients for pairwise comparisons of time-step decoder weights (mean over 80 repetitions) for avoid versus error decoders from Fig. 3c (right, black line). Especially before action onset, decoder weights show high correlations, indicating a stable representation of avoidance-predictive activity. Based on this finding, we trained a single time-independent decoder for all time steps, whose weights we then used to define the Avoid dimension. (**f**) Accuracy of avoid versus error decoding per time step for a set of time-dependent decoders trained individually per time step, and one single time-independent decoder trained using data from all time steps (mean over 80 repetitions). The time-independent decoder was separately evaluated with data from individual time steps. The accuracies before action onset are matched between the two settings (time-dependent and time-independent), suggesting that avoidance-predictive activity can be captured using a single decoder. We therefore used the weights of this single time-independent decoder to define avoid dimensions. (**g**) Decoding accuracy of time-independent decoders for the progressive removal of avoid dimensions (mean and 95% CIs over 80 repetitions). Avoid dimensions are defined by the decoder’s weights vector and are iteratively removed via nullspace projections. Comparison to randomly removed dimensions in blue. (**h**) Analysis of variability over subjects. To assess decoding accuracies per subject we trained a single decoder using data from all subjects but evaluated it separately using data from individual subjects. The resulting accuracies show differences in magnitude but all follow the same temporal dynamics as time-independent decoder trained on pooled data (the green line in **e**, Pearson correlation coefficient 0.984 ± 0.012, mean ± s.d. over 12 subjects). This suggests that the effects captured with our joint analysis of all subjects are representative of effects on the single subject level. (**i**–**l**) Analogous to **e**–**h** for tone decoding. Time-step tone decoders show representational stability (**i**), performance of time-step decoders can be matched using a single time-independent decoder (**j**) and the temporal evolution of decoding accuracies is consistent between pooled data and individual subjects (**l**, Pearson correlation coefficient 0.987 ± 0.015, mean ± s.d. over 12 subjects).

The fact that decoding performance is higher for the avoid setting than the ITI setting suggests that, in addition to the predictive information related to the shuttle motion (present in ITI and avoid settings), there exists predictive information in neural activity that is specific to avoidance actions. We thus hypothesized that the predictive performance of avoid and ITI decoders was based on different population activity patterns. To test this hypothesis, we first used principal component analysis (PCA) to identify dimensions containing motion-related activity as the dimensions of maximal variance during ITI shuttles (Extended Data Fig. 8a–d). Next, we tested how removing these motion dimensions from the joint subspace affected decoding performance in the ITI and avoid settings. We removed motion dimensions by projecting trial data from the joint subspace into the nullspace of the considered motion dimensions. We found that removing two motion dimensions led to the largest relative drop in ITI decoding accuracies and that the decrease in predictive accuracy was substantially lower for avoid versus error decoding (Fig. 3a,b). These results show that most of the motion-related activity is contained in a low-dimensional subspace and that avoidance decoding does not depend on activity in this subspace. Thus, there must be avoidance-specific activity in different dimensions, and we next asked if we could capture these dimensions in the remaining neuronal subspace (that is, the nullspace of the two identified motion dimensions).

To identify avoidance-specific coding dimensions, we devised an iterative approach based on decoding (Extended Data Fig. 8e–h). We first projected all trial data into the motion nullspace (using two motion dimensions) to remove predictive information related to motion. Next, we trained a time-independent SVM decoder to discriminate between avoid and error trials and interpreted the projection axis of the decoder as an avoid dimension. To find additional avoid dimensions, we again projected trial data into the nullspace of the identified avoid dimension and repeated the process. We again evaluated the removal of the identified avoid dimensions for the ITI and avoidance settings and found that the removal of the first two avoid dimensions strongly reduced performance in the avoid versus error setting but not the ITI setting (Fig. 3c,d). Taken together, these results show that it is possible to identify a low-dimensional subspace containing avoidance-specific activity, which is orthogonal to the dimensions containing motion-related activity.

### mPFC population activity can be decomposed into interpretable, orthogonal dimensions

In addition to avoidance and general motion, tone stimuli are a key variable during active avoidance trials. We thus asked if we could identify tone-related activity in the nullspace of the four identified motion and avoidance dimensions (Fig. 3e). We first trained SVM decoders to discriminate between tone (during avoid and error trials) and nontone (during ITI) time periods (Fig. 3f). We found that shortly after tone onset, the decoding accuracy was consistently above 80% (Fig. 3g), indicating the presence of a reliable tone representation during the trial. To investigate the dimensionality of this tone representation, we again tested the effect of iteratively removing tone decoding dimensions. Removing the first dimension decreased the mean accuracy from 79.9% (95% confidence interval (CI) (78.5, 80.9)) to 63.3% (95% CI (57.8, 67.0); Fig. 3h). While this first dimension did not contain all tone-related information, it captured the majority (80.5% (95% CI (65.8, 92.3))) of the remaining variance in the joint coding subspace, whereas subsequent decoding dimensions were limited to 12.6% (95% CI (2.0, 29.0)) or less (Fig. 3h). We therefore focused on this one-tone dimension in subsequent analyses. Taken together, the decomposition of mPFC neuronal activity into five orthogonal dimensions (motion 1, motion 2, avoid 1, avoid 2 and tone) constitutes a compact and interpretable representation of task-related neural activity.

**Fig. 4:**
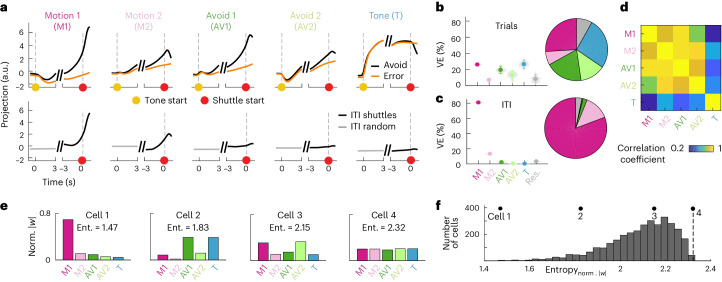
Characterization of low-dimensional task-related population activity. **a**, Mean projections (*n* = 80 repetitions) of neural data onto the five coding dimensions for avoid and error trials (top row) and ITI shuttles and random ITI periods (bottom row). **b**, VE by individual dimensions for avoid and error trials (distributions over 80 repetitions, mean and 95% CIs). **c**, Same as **b** for ITI shuttles. **d**, Pearson correlation coefficient between pairs of coding dimension projections (avoid and error projections concatenated; mean over 80 repetitions). **e**, Weight distributions for four example cells. We calculated how individual cells contributed to the five coding dimensions (Main) and normalized the weight values such that the sum of their absolute values was equal to 1. The following four examples show different types of distributions: (1) selective, (2 and 3) mixed-selective and (4) nonselective. **f**, Distribution of weight entropy values over all recorded cells. Black dots indicate the four example cells from **e**, and the dashed line indicates the maximum possible entropy. Ent., entropy.

**Extended Data Fig. 9:**
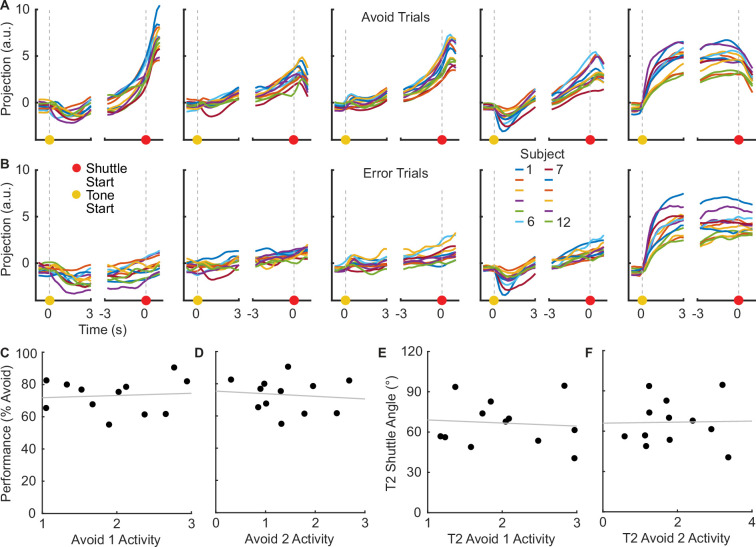
Variability in neural activity over subjects and relation to avoidance behavior. (**a**) Projections of avoid-trial neural activity onto the five coding dimensions (Fig. 4a) for each subject. (**b**) Same as **a** for error trials. (**c**) To test whether variability in neural activity over subjects relates to behavioral variability, we correlated activity in the avoid dimensions to the overall task performance and task 2 shuttle angle over subjects (see following panels). Panel **c** shows the relation of the magnitude of activity in the avoid 1 dimension (time-averaged over the 3 s window preceding avoid start and averaged over all avoid trials) and overall performance (% avoid trials in active avoidance sessions). We found no clear correlation for this or any of the following analyses (Pearson correlation coefficient = 0.07). (**d**) Same as **c** for the avoid 2 dimension (Pearson correlation coefficient = −0.07). (**e**) Relation between magnitude of activity in the avoid 1 dimension during task 2 (time-averaged over the 3 s window preceding avoid start and averaged over all task 2 avoid trials) and the task 2 shuttle angle (see Extended Data Fig. 1d; Pearson correlation coefficient = −0.04). (**f**) Same as **e** for the avoid 2 dimension (Pearson correlation coefficient = −0.02).

To analyze how population activity in the five coding dimensions evolves over the trial, we projected the activity into each of these dimensions (Fig. 4a (top row) and Extended Data Fig. 9). We found that during avoid and error trials, the activity in the two motion and the two avoid dimensions followed similar trajectories (Fig. 4d; Pearson correlation coefficient = 0.88 ± 0.06, mean ± s.d. over six comparisons). Activity in these four dimensions was low at the tone start, with no differences between avoid and error trials. Activity then ramped up toward the start of the avoidance shuttle, with a stronger increase in avoid trials compared to error trials. In contrast, activity in the tone dimension was strongly affected by tone onset and exhibited similar trajectories for avoid and error trials up to shuttle start. Overall, the five coding dimensions captured 91.9% (95% CI (86.0, 96.6)) of the variance, showing that our subspace decomposition did not miss any major sources of activity in the avoid and error trial averages (Fig. 4b). Despite the similarity of the temporal evolution of activity in the motion and avoid dimensions during the trial, there were clear differences between these dimensions for ITI shuttling (Fig. 4a (bottom row)). Activity in the motion dimensions increased around the ITI shuttle start in a similar way to the avoid shuttle start. The two motion dimensions accounted for 94.0% (95% CI (93.1, 95.1)) of the variance in the population activity averaged over ITI shuttles (Fig. 4c). In contrast, the avoid dimensions only explained 2.6% (95% CI (1.7, 3.2)) of the variance, as activity was not strongly affected by ITI shuttles. Taken together, these results show that a substantial fraction of the behavior-related neural variability during avoid trials is not contained in the dimensions that capture motion in the ITI but rather in avoidance-specific dimensions. Nevertheless, within the two motion dimensions, activity is similar between avoid and ITI shuttles, suggesting that these dimensions capture motion irrespective of behavioral context.

To assess how the five coding dimensions relate to the activity of individual cells, we calculated dimension weight vectors for individual subjects by mapping the subject-specific projection matrices from the subject alignment procedure (Extended Data Fig. 6b) onto the five coding dimensions. To quantify how a given cell contributed to the activity in the five coding dimensions, we normalized the five weight values such that the sum of their absolute values was equal to 1 (Fig. 4e). We then calculated the entropy of this distribution over the five dimensions to measure if cells were selective to an individual dimension (low entropy) or contributed to multiple dimensions (high entropy). The distribution of entropy values of all recorded cells shows that the vast majority of cells displayed mixed selectivity to multiple dimensions, while only a few cells were selective to an individual dimension (Fig. 4f). These results suggest that the signals in the identified coding dimensions are carried by a population of mixed-selective cells rather than by different subpopulations coding for individual dimensions.

**Fig. 5:**
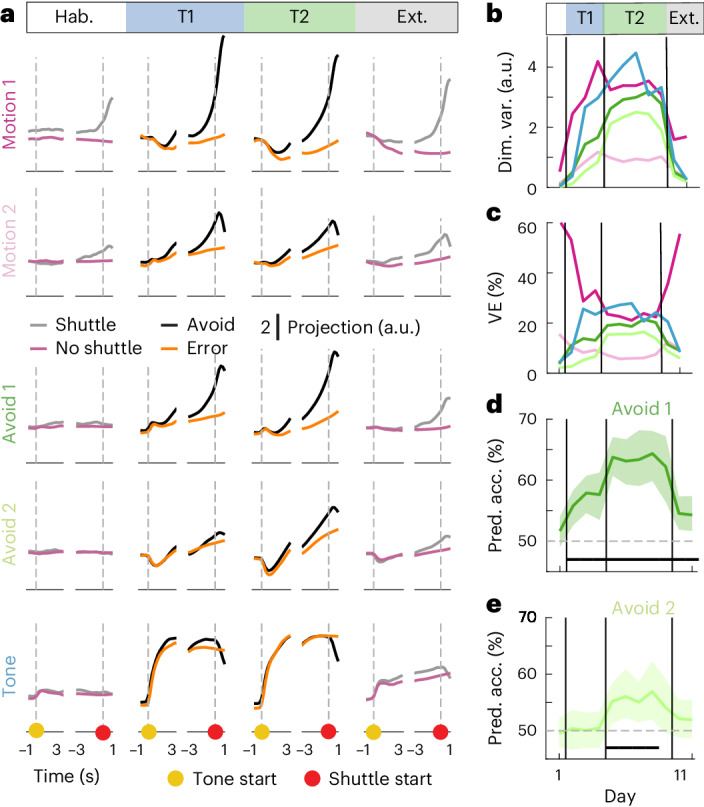
Emergence of low-dimensional, task-related neuronal signals in mPFC. **a**, Mean projections (*n* = 80 repetitions) of neural data onto the five coding dimensions for trials with and without shuttling during habituation (day 1), task 1 (days 2–4), task 2 (days 5–9) and extinction (days 10–11). **b**, Absolute variance within each coding dimension. **c**, Relative VE by the five coding dimensions across the 11-day learning paradigm. Mean over 80 repetitions. **d**, Avoid versus error decoding accuracy (time-averaged for the 2 s preceding shuttle start) for the decoder that was used to define the avoid 1 dimension (mean and 95% CIs for 80 repetitions). Decoders were trained with data from tasks 1 and 2 and separately evaluated with test data from individual days. The black bar indicates performance that is significantly above chance based on 50% not being included in the CI. **e**, Same as **d**, but for the decoder that was used to define the avoid 2 dimension (the avoid 1 dimension was already removed). T1, task 1; T2, task 2.

**Extended Data Fig. 10:**
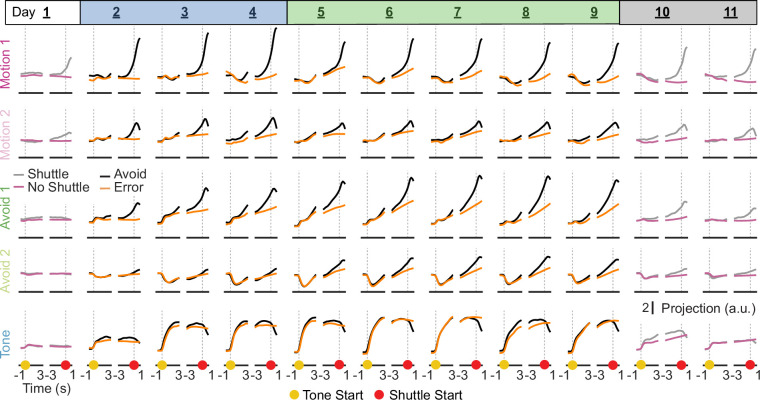
Coding dimension projections per session. Mean projection (n = 80 repetitions) of the five coding dimensions (as presented in Fig. 4) displayed across all 11 days of learning for trials with and without shuttling.

We next asked, how the activity in the five-dimensional coding space evolved over our learning paradigm by analyzing projections calculated for different phases of the experiment (Fig. 5a–c and Extended Data Fig. 10). Motion-related activity dominated the neural variability in habituation and extinction sessions but had a reduced relative contribution during active avoidance sessions (63.3% (95% CI (59.4, 67.4)) versus 36.1% (95% CI (35.0, 37.0)) variance explained (VE); Fig. 5c). In contrast, tone-related and avoidance-specific activity emerged in active avoidance sessions (51.4% (95% CI (46.6, 55.0)) VE versus 10.1% (95% CI (5.8, 16.1)) in habituation; Fig. 5c), indicating that these activity patterns are learned and task-related. These results show that mPFC activity is engaged during active avoidance learning and develops responses to behaviorally relevant sensory stimuli as well as activity specific to avoidance actions.

### Avoidance-specific activity distinguishes between tasks

The avoid dimensions seemed to be differently engaged in tasks 1 and 2, suggesting a task-related change in avoidance-specific activity (Fig. 5a–c). Based on this observation, we further investigated the avoid versus error decoders that we initially used to define the avoid 1 and avoid 2 dimensions (Fig. 3 and Extended Data Fig. 8). To assess time-dependent changes in the decoders’ ability to discriminate avoid and error trials, we trained the decoders using data from all avoidance sessions but tested them using data split into individual sessions (Fig. 5d,e). We found that the avoid 1 decoder worked best in task 2 sessions but also showed above chance performance in task 1 (Fig. 5d). In contrast, the avoid 2 decoder performed above chance level in task 2, but not in task 1 (Fig. 5e). This difference in decoding performance indicates that avoid 1 activity generalizes to both avoidance behaviors, while avoid 2 emerges with the task switch to accommodate the altered avoidance behavior in task 2. Taken together, these results suggest that the task switch changes the mPFC coding of the avoidance action by layering additional avoidance-specific activity.

**Fig. 6:**
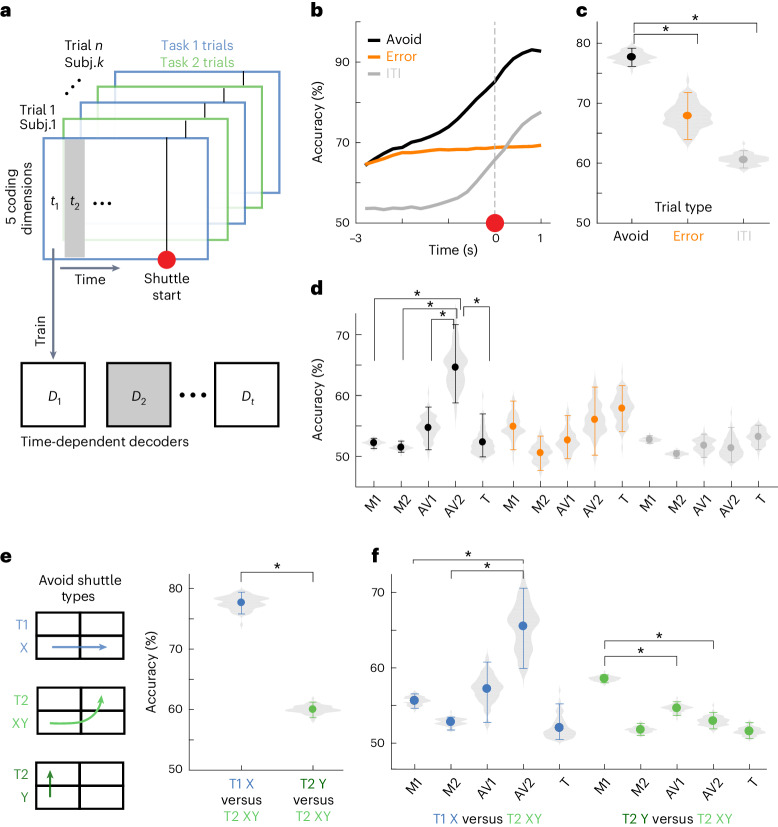
The task switch affects avoidance population coding. **a**, Schematic representation of task decoding, where we trained decoders to distinguish data from tasks 1 and 2 trials. **b**, Task-decoding accuracy for time-dependent decoders trained to discriminate between task 1 and task 2 data separately for avoid trials, error trials or ITI shuttles (mean over 80 repetitions). **c**, Temporal average (−3 s to 1 s) for data from **b** (mean and 95% CIs for 80 repetitions). Significant differences are reported based on nonoverlapping CIs. **d**, Time-averaged task-decoding accuracy (−3 s to 1 s) for decoding performed with individual dimensions for avoid trials, error trials and ITI shuttles (mean and 95% CIs for 80 repetitions). **e**, Decoding of different types of avoid trials. T1 X shuttles and T2 XY shuttles differ in motion and task, and T2 Y shuttles and T2 XY shuttles also differ in motion but follow the same task rule. Time-averaged accuracy (−3 s to 1 s) for decoders using all five coding dimensions (mean and 95% CIs for 80 repetitions). **f**, Shuttle-type decoding, as in **e**, for decoders trained on individual dimensions. The single asterisk denotes significance derived from nonoverlapping CIs (Software and Statistics and reproducibility).

To test whether the task-related changes were specific to the avoid 2 dimension or also affected other dimensions, we explicitly tested for task-based differences using an additional decoding analysis. We first trained decoders to discriminate trial data from tasks 1 and 2 (task decoding; Fig. 6a), analogously to avoid versus error decoding. We trained independent sets of time-dependent task decoders for avoid trials, error trials and ITI shuttles (X shuttles in task 1 and Y shuttles in task 2) based on the activity in the five-dimensional coding space and found that task-decoding accuracy differed between the three settings (Fig. 6b,c). Task decoding was more accurate for avoid trials than for error trials or ITI shuttles (Fig. 6c). During avoid trials, decoding accuracy ramped up toward avoidance actions (Fig. 6b). These dynamics were less pronounced on error trials, indicating that the task switch did not affect task-related neural activity in general, but specifically altered the neural dynamics related to the execution of avoidance actions. Although task-decoding accuracy for ITI shuttles also increased toward the shuttle action, the performance was generally lower than for the avoid setting. This suggests that task-decoding in avoid trials was predominantly based on avoidance-specific rather than motion-related activity.

To further investigate how task-specific information was distributed, we next trained individual task decoders for the five coding dimensions. We found that the avoid 2 dimension achieved the highest task-decoding accuracies in the avoid, but not in the error and ITI settings (Fig. 6d). These results show that the task-related change in avoidance behavior is associated with a change in avoidance-specific activity, suggesting that the updated neural dynamics in mPFC could be the basis of the change in behavior.

The task switch alters multiple aspects of the behavior—the direction of the shuttle motion (that is, a physical feature of the behavior) and the relation between behavior and trial outcome (that is, an abstract feature of the behavior determined by task design). We therefore next asked how the change of these two aspects of the behavior relates to the observed change in neural activity. To address this question, we made use of the behavioral variability of task 2 avoid trials (Fig. 6e,f). In task 2, avoidance only requires motion in the *y* dimension and is independent of motion in the *x* dimension. However, animals frequently performed shuttles that crossed both the *x* and *y* midlines (XY shuttles). Of all of the 1,624 task 2 avoid trials, 930 were Y shuttles (57.3%) and 694 were XY shuttles (42.7%). In terms of their motion kinematics, task 2 XY shuttles differ from both task 1 X shuttles and task 2 Y shuttles. In the abstract view of the task design, however, they only differ from task 1 X shuttles but not from task 2 Y shuttles. To test how this was reflected in the activity in the identified coding dimensions, we again trained decoders to distinguish between the different shuttle types. We found that decoders were substantially more successful in distinguishing task 2 XY shuttles from task 1 X shuttles than from task 2 Y shuttles (Fig. 6e). We also found that, for the task 1 X shuttle versus task 2 XY shuttle setting, the avoid 2 dimension carried more information than any of the motion dimensions (Fig. 6f (left)). In contrast, for the task 2 Y shuttle versus task 2 XY shuttle setting, the motion 1 dimension contained more information than any of the avoid dimensions (Fig. 6f (right)). Taken together, these results suggest that the task-related difference in the avoid 2 dimension (Fig. 6f) cannot solely be explained by differences in motion and are thus based on the abstract task-related difference between the two actions.

### mPFC sensory responses are modulated by avoidance behavior

**Fig. 7:**
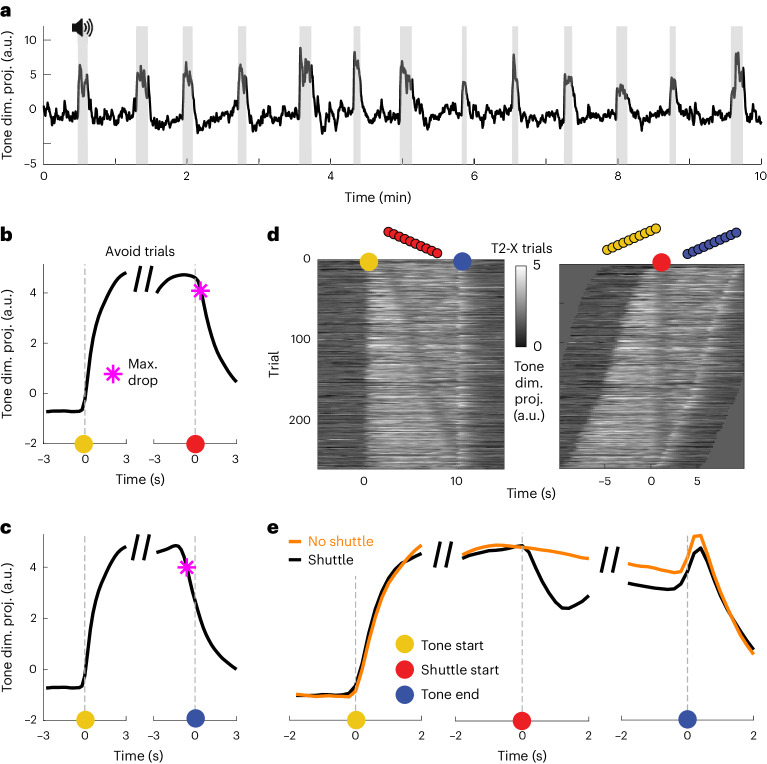
Avoidance behavior affects mPFC tone encoding. **a**, Tone dimension projection over a 10-min time window from an example session of one subject. Tone presentations are marked in gray. **b**, Mean projection (*n* = 80 repetitions) of neural data onto the tone dimension during avoid trials aligned to tone start (left) and shuttle start (right). The maximum drop point (purple star) refers to the time step before the maximum decrease of tone dimension activity between two consecutive time steps (5 Hz). **c**, Same as **b**, but aligned to tone start (left) and tone end (right). **d**, Tone dimension activity for task 2 X-shuttle trials (error trials with an incorrect shuttle). Trials were either aligned to the tone start (left) or shuttle start (right). **e**, Trial averages for data from **d** (black line) aligned to tone start (left), shuttle start (middle) and tone end (right). The orange line represents trials without shuttles. For these trials, the shuttle start point was randomly sampled to match the distribution of shuttle starts from the shuttle trials.

Our subspace decomposition analysis shows that tone-related and avoidance-specific activity can be decomposed into independent dimensions. Yet, we also observed that the activity in the tone dimension was modulated by the execution of avoidance actions (Fig. 7). In general, tone dimension activity was well correlated with the binary tone on/off timing for individual subjects (Fig. 7a; Pearson correlation coefficient = 0.62 (95% CI (0.60, 0.63)), average over subjects and active avoidance sessions, mean and CI over 80 repetitions). However, we observed an exception at the time of shuttle start, where the tone signal dropped two time steps (400 ms) after shuttle start (Fig. 7b), although the tone only turned off approximately 1 s after shuttle start when the action was completed (Fig. 1h). Alignment to the end of the tone showed that the drop of activity in the tone dimension occurred three time steps (600 ms) before the actual offset of the tone (Fig. 7c). To further examine the interaction between the tone dimension and the execution of the tone-induced shuttle behavior, we next focused on a particular trial set from the transition period between tasks 1 and 2. In early task 2 trials, mice performed X shuttles as learned in task 1, which, however, did not lead to avoidance anymore in task 2. During these task 2 X shuttles, we observed a similar drop in the tone dimension activity aligned to action onset, despite the continued tone presentation (Fig. 7d,e). At 1.2 s after action onset, the tone dimension activity was decreased by 36.7% (95% CI (28.8, 43.5)) as compared to trials without shuttle actions. These results suggest that the mPFC tone representation is modulated by the execution of the learned behavior that has been associated with the termination of the tone and the avoidance of the shock.

## Discussion

In this study, we developed a new two-dimensional active avoidance paradigm and combined it with large-scale neural recordings in mouse mPFC and a new data analysis approach. This allowed us to identify and characterize mPFC neural correlates of sensory stimuli and avoidance actions and to study them over learning. We show that the recorded high-dimensional population activity can be decomposed into five interpretable orthogonal dimensions encoding motion, tone and avoidance. Notably, our approach allowed us to distinguish between learned avoidance-specific activity and activity related to general motion. We show that these signals exhibit similar dynamics during active avoidance trials but behave differently during the ITI. In addition, we found that activity in tone and avoidance dimensions emerges with learning and disappears again in extinction sessions, consistent with a model in which mPFC uses sensory-driven responses to drive behavior execution. Moreover, one of the identified avoidance dimensions discriminated between the two avoidance tasks and only emerged in the second task. This suggests that the mPFC represents behaviors with sufficient resolution to enable linking stimuli to specific behavioral responses. Interestingly, we found that the execution of avoidance behaviors suppressed sensory-related activity, suggesting that mPFC sensory representations also depend on the behavior of the animal. Overall, these results point toward the mPFC implementing the sensory-behavior link through dynamically interacting neural correlates that represent essential task features and are contained within a low-dimensional subspace of the overall population activity.

The interpretation of neural activity during active avoidance trials is challenging due to the temporal overlap of sensory stimuli, cognitive processes and motor signals, as well as mixed selectivity to these signals. We addressed these challenges by combining several data analysis steps that allowed us to identify and isolate distinct and well-defined neural correlates at the population level. First, we used a procedure to align the neural responses recorded from different subjects into a joint coding subspace^19,20^. This allowed us to jointly analyze all recorded trials and to use SVM decoders to accurately identify the subspace dimensions that contained avoidance-specific (Fig. 3c) and tone-related activity (Fig. 3g). An often-used and powerful alternative for relating neural activity to task variables is the use of regression-based approaches^17,21,22^. However, in the setting of multiple temporally correlated predictor variables (such as motion, avoidance and tone), it becomes challenging to specify a regression model that properly isolates these variables. Our decomposition approach allowed us to sequentially identify meaningful dimensions using suitable decoding settings, and the nullspace projections ensured that the resulting coding space isolated individual features in orthogonal dimensions (Fig. 3e).

While previous work already showed that mPFC activity contains avoidance-predictive information^8^, our approach allowed us to identify and characterize the activity patterns that carry this information. The fact that we could identify avoidance-specific activity patterns that were not present during ITI shuttles (Fig. 4a) indicates that these activity patterns resulted from the processing of tone stimulus information. Nevertheless, we found that a large fraction of tone-driven activity was independent of the execution of avoidance shuttles (tone dimension; Fig. 4a). This suggests that whether or not an animal performs an avoidance action is not based on differences in sensory input to mPFC but depends on mPFC’s processing of the incoming sensory information. Furthermore, the activity in the identified coding dimensions was not based on distinct subpopulations coding for individual variables but rather on a population of cells showing diverse forms of mixed selectivity (Fig. 4e,f). This high degree of mixing is consistent with previous work, which showed that mPFC responses in an approach-avoidance task show higher degrees of mixed selectivity than basolateral amygdala (BLA) responses^23^. This mixed selectivity goes along with a higher representational capacity that may be necessary for behavioral flexibility^23,24^. Taken together, our results are consistent with a model in which sensory-driven mPFC responses partake in a distributed dynamical process^25^ to drive behavior execution.

While our results are only correlational, multiple studies have demonstrated the causal role of mPFC in active avoidance^7,8,26,27^. A recent study showed that mPFC’s influence on avoidance behavior is mediated by projections to the BLA and nucleus accumbens^7^. Additionally, Kajs et al.^28^ used fiber photometry recordings to show that populations of cells projecting to the BLA and the striatum differentially encode avoidance actions. Such projection-specific differences in mPFC activity have also been shown to be important for various other tasks^3,14,29^. How the high degree of mixed selectivity we observed at the single-cell level maps onto such activity differences in projection-specific subpopulations remains to be studied^30^.

In our study, we only record from excitatory cells, but inhibitory activity is crucial to understanding the transformation from stimuli to behaviors in mPFC. For example, mPFC inhibitory signals are required for avoidance^26^, and in fear conditioning, mPFC inhibitory neurons are important for temporally structuring the activity of pyramidal neurons^4,6^. Furthermore, specific interneuron types encode different task-related signals^31^, and it will be interesting to see how these are related to the diversity of responses of the pyramidal cells we report in this study.

Our result that activity in the tone dimension is modulated by behavior execution (Fig. 7) indicates that tone-driven mPFC signals are not purely sensory but are modulated by the behavior of the animal. The drop in tone-driven activity at action onset, despite continued sensory input, indicates a change in information flow induced by the execution of the learned avoidance action. However, it is unclear what causes the observed drop in activity. A recent study demonstrated the learned suppression of auditory cortex activity in response to movement-related sounds through inhibition via motor cortex inputs^32^. In mPFC, another potential substrate for the observed tone signal dynamics is the bidirectional interaction with the BLA. mPFC tone responses are dependent on inputs from the BLA^8^. Furthermore, the BLA is generally required for avoidance learning^33^ but is also involved in the expression of avoidance behavior^7,34^. These results highlight the complex interaction between sensory processing and behavior execution, and further work is needed to understand the temporal dynamics of sensory information flow through the network of involved brain areas.

Finally, the switch between the two active avoidance actions (X and Y shuttling) allowed us to study behavioral flexibility in mPFC. mPFC has previously been shown to be involved in switching between tasks or rules^35–37^, and our results offer new insights into how behavior-related neural activity is updated upon a switch between conditioned behavioral responses. We found that avoidance-specific activity was organized into two dimensions, where one was general to both avoidance behaviors (avoid 1) and the other was specific to shuttling along the *Y* dimension and only emerged in task 2 (avoid 2; Figs. 5 and 6). Notably, our analysis of X, Y and XY shuttles (Fig. 6e,f) demonstrates that the change in the avoid 2 dimension cannot be explained by the mere change of the shuttle direction but is instead a more abstract reflection of the changed task contingency and the required update of the learned sensorimotor transformation. The sequential layering of previously learned transformations and the newly added dimensions might help animals not only to maintain the memory of previously learned tasks but also to shift between tasks in a context-dependent manner. In fact, the similar temporal dynamics of activity in motion and avoidance dimensions (Fig. 4a,d) could indicate that, with progressive learning, new correlates of avoidance behavior are derived from either naive or previously learned behavioral primitives. The high level of mixed selectivity in mPFC should greatly facilitate such layered learning, and future work should investigate how context-specific recombination of sensory and behavioral neural correlates might facilitate behavioral flexibility.

## Methods

All animal procedures and experiments were approved by the Cantonal Veterinary Office in Zurich, Switzerland.

### Subjects

All experiments were performed on male C57Bl6/Crl1 mice (Charles River Laboratories) aged between 4 and 7 months at the start of the behavioral experiment. Animals were housed in individually ventilated cages in a 12-h light/12-h dark cycle room (lights on from 6:30 to 18:30, ambient temperature: 21–24 °C, humidity: 35–70%) and were provided food and water ad libitum. After import from the breeders, mice were given a 2-week acclimatization period to the new housing condition before the first surgery. During the experiments, mice were kept in groups of two to five animals.

### Surgical procedures

#### Anesthesia

For all procedures, including anesthesia, mice received pre-emptive buprenorphine (Bupaq; Streuli, 0.1 mg kg^−1^) 20–30 min before anesthesia. Anesthesia was induced with a Ketamine–Xylazine cocktail (Ketanarcon; Streuli, 90 mg kg^−1^/Xylazin; Streuli, 8 mg kg^−1^), and mice were mounted onto a stereotactic frame (Kopf Instruments). During the procedure, mice received 95% medical O_2_ (PanGas, Conoxia) through a face mask, and their body temperature was kept steady at 37 °C using a temperature controller and a heating pad.

#### Viral injections

At the time of the first surgery, mice were 8–13 weeks old. To label excitatory neurons in the prelimbic cortex, we intracranially injected 500 nl (titer: 4 × 10^11^) of an adeno-associated virus driving the expression of GCaMP6m via the CamKII-promoter (AAV2/5-CamKIIa-GCaMP6m) into the prelimbic cortex (anterior-posterior, 1.8; medial-lateral, 0.4; dorsal-ventral, 2.1). We used either a micropump (UMP3 UltraMicroPump; World Precision Instruments) or a borosilicate glass pipette with a 50 μm diameter tip and injected the virus by applying short pressure pulses at a speed of approximately 100 nl min^−1^. After injection, the needle/glass pipette was left in place for 5 min to avoid backspill. Finally, the skin was closed using surgical sutures.

#### Microendoscope implantation

A total of 7–14 days after the viral injection, we implanted a small stainless steel guide tube (1.2 mm diameter; Ziggy’s tubes and wires) with a custom glass coverslip (0.125-mm thick BK7 glass; Electron Microscopy Sciences) glued to one end as previously described in ref. ^38^. In brief, we first made a 1.2 mm diameter (round) craniotomy centered above the ventral-mPFC (1.8 mm anterior, 0.4 mm medial, relative to bregma). To avoid increased intracranial pressure when inserting the implant, we aspirated tissue down to a depth of 1.9 mm from the skull surface. Next, we lowered the guide tube to the bottom of the incision (2.2 mm relative to the skull surface) and glued the guide tube to the mouse skull using ultraviolet-curable glue (4305 LC; Loctite). We then applied dental acrylic (Metabond; Parkell or Scotchbond ESPE; 3M) over the complete cranium and around the guide tube. Finally, we attached a metal bar and applied dental acrylic cement (Paladur) to stabilize the implant.

#### Analgesic regime

For 3 days after each surgical procedure, animals received buprenorphine subcutaneous (Bupaq; Streuli, 0.1 mg kg^−1^) every 6 h during the light cycle and in the drinking water (Bupaq; Streuli, 0.01 mg ml^−1^) during the dark cycle, as well as carprofen subcutaneous (Rimadyl; Zoetis, 4 mg kg^−1^) every 12 h.

#### Preparation of animals for behavioral experiments

Animals received 6–12 weeks of recovery time before testing viral expression levels. Approximately 1 week before starting behavioral experiments, we inserted the gradient index (GRIN) lens into the guide tube (GT-IFRL-100-101027-50-NC; Grin Technologies) and attached a microscope base plate (Inscopix) above the implanted microendoscope with blue light-curable glue (Flow-it; Pentron).

### Validation of imaging methodology

#### Perfusion

After completion of experiments, animals were given terminal anesthesia with pentobarbital (Esconarkon; Streuli, 200 mg kg^−1^) and perfused transcardially with PBS followed by 4% paraformaldehyde (PFA). Brain tissue was removed and postfixed for 24–48 h in 4% PFA. Coronal slices (50-μm thick) were prepared on a vibratome (VT1000 S; Leica) and stored in PBS.

#### Verification of microendoscopic implant

To confirm the placement of the GRIN lenses in the mPFC, cyto-structural differences in the tissue were highlighted using Nissl stain (NeuroTrace 530/615; Invitrogen) following the provided protocol from Invitrogen with a dilution of 1:50 NeuroTrace. Slices containing the prefrontal cortex were mounted, and images were acquired using a fluorescence microscope (Olympus, BX51). Images were overlaid using the reference pictures from ref. ^39^. For each section, we marked the position of the base of the microendoscope for every mouse (Extended Data Fig. 2b).

#### Verification of cell type

Standard immunofluorescence protocols were used to stain inhibitory and excitatory neurons. Slides were incubated with the primary antibody (either rabbit anti-Neurogranin (Millipore, 07-425; 1:2,000) or rabbit anti-GAD65 (Millipore, AB1511; 1:500)) at 4 °C overnight followed by a 2-h incubation at room temperature with the secondary antibody Alexa 594 anti-rabbit (Invitrogen, A-11062; 1:200). Slides were further stained for 4 min with DAPI (Invitrogen, D1306; 1:1,000) in PBS (0.1 M) before mounting. Confocal pictures were taken in red (at wavelength 594 nm; Neurogranin or GAD65), green (at wavelength 488 nm; GCaMP6m) and blue channels (at wavelength 390 nm; DAPI), and pictures were compared for overlap of labeling (Extended Data Fig. 2c,d; acquired with Leica Stellaris 5, LAS X software).

### Behavioral procedures

#### Calcium imaging during mouse learning behavior

Calcium imaging experiments were performed using a miniaturized fluorescence microscope (nVista HD 2.0; Inscopix). Before behavioral experiments, we habituated all mice to the mounting procedure and the weight of the miniscope for at least three consecutive days. During the mounting procedure, animals were briefly head-fixed by fixing their metal head bar to a custom-made mounting station with a running disk. Additionally, subjects were habituated to the experimental room and were handled by the experimenter for 5 of 7 days preceding the experiment. In every imaging session, we verified for absence of shifts in the field of view and slightly adjusted the microscope focus if necessary. We acquired frames of 1,000 × 1,000 pixels at 12 bits and a frame rate of 20 Hz. To acquire the calcium imaging data, we used a light-emitting diode intensity between 10% and 25% (100–150 μW) depending on the strength of the GCaMP6m expression. For all recordings, we used the maximum imaging sensor gain level of 4. All recorded data were directly streamed to the hard disk of a desktop computer.

#### Two-dimensional active avoidance

For the two-dimensional active avoidance experiments, we used a rectangular shuttle box (Cambridge Instruments), which we separated into four compartments by using four equally sized platforms. We 3D-printed these movable platforms to fit between the bars of the shock grid, which allowed us to dynamically adjust the safe zone during training. In the default position, the platforms were situated below the shock grid such that mice could not jump onto them to avoid contact with the grid. In the elevated state, mice could fully stand on the platform without being in contact with the shock grid, thereby creating the possibility of escaping shocks. We controlled the platforms using servo motors that we placed outside of the isolation chamber. The complete learning paradigm had a duration of 11 days, comprising habituation (day 1), active avoidance task 1 (days 2–4), active avoidance task 2 (days 5–9) and extinction sessions (days 10 and 11). All sessions had a duration of 40 min and contained 50 trials with pseudorandom ITIs of 30 ± 10 s. Each of the trials started with the presentation of an 8 kHz tone at 80 dB for 10 s. In all active avoidance sessions (days 2–9), the tone was followed by a light foot shock (0.2 mA) with a maximal duration of 5 s. For each of the trials, we defined half of the shuttle box as a safe zone. We determined the position of the safe zone by the trial type (task 1 or task 2) and the position of the animal at the start of the trial. For task 1 trials, mice had to cross the midline along the *x* axis (X shuttles) of the cage to reach the safe zone, whereas for task 2 trials, mice had to cross the midline along the *y* axis (Fig. 1a). If mice entered the safe zone during tone or shock presentation, we blocked both tone and shock channels until the end of the trial. If mice did not shut off the tone before shock onset, we elevated the two platforms in the safe zone for a duration of 15 s, time-locked to the onset of the shock, providing mice with the possibility to escape. We recorded all mouse behavior using two top-view B/W cameras (DMK 23FV024; ImagingSource) which covered the entire cage and were later merged to produce a single behavior video. The recording of individual frames of the behavior cameras was synchronized to the miniscope recordings using a hardware trigger, which allowed the exact alignment of neural and behavioral data.

### Extracting neural activity from calcium imaging data

#### Preprocessing of calcium imaging data

We implemented the following procedures to preprocess the video of each individual imaging session. We first spatially downsampled all frames by a factor of 2 to obtain 500 × 500-pixel frames. Next, we used the TurboReg algorithm^38^ for motion correction by aligning each frame to a reference frame. We then temporally downsampled videos by a factor of 4, resulting in a frame rate of 5 Hz. To account for slow changes in luminosity related to bleaching, we fit a rank-2 bleaching model by running PCA on a temporally smoothed version of the video and then subtracting this model from the original video. Next, to remove wide-field luminosity fluctuations occurring on a faster time scale (for example, neuropil signals), we normalized each frame by dividing it by its lowpass-filtered version (using a Gaussian spatial frequency filter with a s.d. of 7; Extended Data Fig. 3c–e and Supplementary Video 3). Finally, we re-expressed all frames in units of relative changes in fluorescence, given by ∆*F*(*t*)/*F*0 = (*F*(*t*) − *F*0)/*F*0, where *F*0 is the mean frame obtained by averaging over the entire movie.

#### Cell extraction for individual sessions

To automatically identify individual neurons in the calcium imaging movies of a given imaging session, we used a well-established cell extraction algorithm based on PCA and independent component analysis (ICA)^39^. This algorithm generates spatial filters that correspond to the cells’ locations, which allowed us to extract the corresponding temporal activity traces. However, instead of extracting these activity traces for each session individually, we first use the positional information contained in the identified spatial filters to align the movies from all imaging sessions of a given mouse.

#### Session alignment

To be able to track cells across imaging sessions, we applied the following alignment procedure for each mouse. We first constructed cell maps for every session by calculating the maximum projection of all cells’ spatial filters onto one image (see outlines in Fig. 1f). We then used MATLAB’s imregister function to align the sessions’ cell maps onto one reference session. We controlled the quality of the alignment by quantifying the pairwise similarity between the cell maps of individual sessions (Extended Data Fig. 3b) and by visually inspecting the alignment (Supplementary Video 4). Based on these criteria, we excluded sessions for which we could not find a satisfactory alignment (Extended Data Fig. 3b). Next, we used the registration coordinates of the aligned cell maps to align all session movies into a common reference frame. This allowed us to concatenate all session movies to construct one movie containing the full experiment. To account for differences in the signal-to-noise ratio of individual sessions, we calculated the overall s.d. of all pixels for every session and then scaled the corresponding movies to match the minimal s.d. The resulting concatenated movie thus contained Δ*F*/*F* values with a stable mean and s.d. over all sessions.

#### Joint analysis of multiple sessions

We used the aligned and concatenated movies of individual subjects and PCA/ICA to obtain spatial filters and activity traces over the whole experiment. Because the high number of frames made running PCA/ICA on the whole concatenated movie intractable, we instead generated spatial filters by performing signal extraction on a reduced movie, containing 6,000 consecutive frames from every session (that is, half of the data). We then recovered the activity traces over the full duration of the concatenated movie by projecting the full movie onto these spatial filters.

#### Postprocessing and validation

A known issue with PCA/ICA is that individual cells are occasionally split into multiple components. To make sure we do not include split cells in our analyses, we detected pairs of cells that have highly correlated activity (Pearson correlation > 0.7) and are spatially close (centroid distance < 20 pixels) and excluded one of the cells for each pair. Finally, we manually validated each cell by inspecting its morphology, activity trace over all sessions, mean calcium transient and checking whether peaks in the activity trace were consistently caused by the same pixel pattern (Extended Data Fig. 4).

### Quantification and statistical analysis

#### Behavior analysis

To analyze animal behavior, we first stitched the videos of the two behavior cameras to obtain a single video. We then used DeepLabCut software^40^ to track five points of the animal (Extended Data Fig. 1). To quantify the overall speed of the animal, we averaged the positions of the three most stable points (left ear, right ear and miniscope bottom) and calculated the instantaneous speed per time step.

#### Alignment of trials and ITI shuttles

We aligned avoid trials according to the start of the avoidance shuttle (shuttle start). We defined the shuttle start time as the timepoint with the maximal increase in instantaneous speed within the 2 s window before the detected shuttle. For all analyses that considered the window starting 3 s before the shuttle start, we discarded avoidance trials with a shuttle start earlier than 3 s after tone start. To ensure that error trials were comparable to avoid trials in terms of trial lengths, we randomly sampled error trial alignment points (pseudoshuttle start) such that they matched the distribution of shuttle start time points of avoid trials between 3 s and 9 s. To account for the variability introduced by this sampling, we repeated each analysis over multiple repetitions (Avoid and error decoding). We aligned ITI shuttles analogously to avoid trials.

#### Single-cell analysis

To define cells as trial-responsive, we considered the 3 s window after tone start and the 3 s window before shuttle start. We calculated *z* scores per time step, using the 6 s window before the trial start as a baseline period. We defined cells as trial-responsive if their mean absolute *z* score exceeded a value of 1.96 (*P* < 0.05, two-tailed; data distribution was assumed to be normal, but this was not formally tested)^8^.

#### Subject alignment

To align the population activity of different subjects into one common subspace, we first collected event-aligned trial averages (Extended Data Fig. 6). We separately aligned data from avoid trials, error trials and the ITI for the two tasks (that is, 2 × 3 conditions). For avoid trials, we used windows around the tone start (−1 s to 3 s) and shuttle start (−3 s to 1 s) alignment points. For error trials, we used the same structure using the sampled alignment points (pseudoshuttle start). For ITI shuttles, we used the window from −4 s to 4 s around shuttle start. We next computed condition averages for each cell in each of the six conditions and concatenated all cells from all animals to obtain six (*n* × *t*) matrices, where *n* is the total number of neurons and *t* is the number of time steps (8 s at 5 Hz). We then mean-subtracted these six matrices and normalized them to have a Frobenius norm of 1. Next, we concatenated the six normalized condition average matrices along the time dimension to obtain an (*n* × 6 *t*) matrix (Extended Data Fig. 6b (left)), on which we then performed PCA. We defined the resulting (*n* × *k*) matrix of coefficient values as the joint subspace (Extended Data Fig. 6b (middle)), where *k* is the number of PCs we chose to use. To compute subject-specific projection matrices for projecting cellular activity into this joint subspace, we split the coefficient matrix along the cell dimension back into coefficient matrices for the individual subjects (Extended Data Fig. 6b (right)). Because these matrices are not orthogonal anymore, we used the QR decomposition to orthogonalize them as the final step of the procedure. To ensure that the alignment procedure did not introduce artifacts in further analyses, we used half of the trials for alignment and the other half for the decoding analyses described below. The choice of trials was randomly assigned for every repetition. We chose to work with a ten-dimensional joint subspace, as ten constitutes a good tradeoff between explained variance and alignment quality (Extended Data Fig. 6d–f). Another factor that we took into account is that our decomposition approach (see below) requires a certain dimensionality to separate neural signals into task-related dimensions. In our analyses, we consider five task-related dimensions, but we empirically observed that a higher number of dimensions in the initial joint subspace led to cleaner separation of signals into these five dimensions, as the decomposition procedures had a higher degree of freedom.

#### Avoid and error decoding

We aligned avoid and error trials (as described above) and trained individual decoders for every time step from −3 s to 1 s from the alignment points. Decoders were linear SVMs with a box constraint parameter of 1, and we used fivefold cross-validation to estimate test accuracies. We used avoid and error trials from days 3–9 and balanced the two classes by subsampling 300 trials per class in all settings. For some avoid trials, the tone can be turned off in the 1 s after shuttle start (Fig. 1h). Because we want the decoders to only capture avoidance-related information, we excluded these trials for the respective time steps to ensure that there is no confounding tone-related information. To deal with the variability introduced through sampling (error trial alignment and trial samples), we repeated each analysis multiple times (typically 80 times, if not reported otherwise) and computed average accuracies over repetitions. When separately evaluating decoders for individual subjects (Extended Data Fig. 8h,l) or sessions (Fig. 5d,e), we split all trials into one training set and one test set (instead of using fivefold cross-validation). This required decreasing the number of trials used for training and testing to 150 per class.

#### ITI control and video decoders

In the ITI decoding setting, we considered the window from −3 s to 1 s around the shuttle start for each ITI shuttle. We trained decoders to discriminate ITI shuttles from random 4 s periods in the ITI. For the video decoder control setting, we used the five-dimensional speed vector from the DeepLabCut tracking points (Extended Data Fig. 1a). The purpose of the ITI control is to assess how much of the effect we see in avoid versus error decoding (Fig. 2c) can be explained by motion-related information. We thus had to match the amount of motion-related information between the avoid versus error and the ITI shuttle versus ITI random settings. We achieved this by selecting ITI shuttles based on their mean speed (faster shuttles are easier to decode; Extended Data Fig. 7a–d). By choosing the ten fastest shuttles from every session for decoding, we could match the performance of video decoders between the avoid and ITI settings (Fig. 2d and Extended Data Fig. 7c,d). This allowed us to conclude that the differences we observed for neural decoders (Fig. 2c) are not based on simple differences in motion.

#### Identification of motion dimensions

To identify motion-related dimensions in the joint coding subspace, we collected ITI shuttles from task 1 and task 2 sessions and computed average activities for the window from −4 s to 4 s around shuttle start. We then performed PCA on the resulting activity matrix and considered the first five PCs as motion dimensions.

#### Identification of avoidance dimensions

To define avoid dimensions, we first projected all trial data into the nullspace of the first two motion dimensions. Next, we iteratively defined avoid dimensions using the following procedures: (1) we train a time-independent avoid/error decoder using randomly sampled time points from the −3 s to 1 s window around shuttle start (one per trial), (2) we compute the avoid dimension by normalizing the decoder weight vector to have a norm of 1 and projecting this vector back from the nullspace into the ten-dimensional subspace, (3) we project the trial data into the nullspace of the space given by the first two motion dimensions and all avoid dimensions and (4) we repeat the process with different trial samples and timepoint samples until we have obtained five avoid dimensions.

#### Identification of tone dimension

To identify tone dimensions, we followed the same strategy as for avoid dimensions in the nullspace of the first two motion dimensions and the first two avoid dimensions. We trained a time-independent tone decoder using randomly sampled time points from the first 5 s of avoid trials (we only used time steps before shuttle start) and error trials. The baseline period was defined using data points from the period 1 s before tone start.

#### Task decoding

We trained time-dependent SVM decoders to discriminate between data from task 1 (days 3 and 4) and task 2 (days 6–9) based on activity in the five-dimensional coding space (Fig. 6a–c). We trained an independent set of decoders for avoid trials, error trials and ITI shuttles. For ITI shuttle task decoding, we used only X shuttles for task 1 and only Y shuttles for task 2, such that the ITI shuttle setting has the same task-specific motion profiles as the avoid setting. This choice ensures that performance differences between the avoid and ITI settings cannot be explained by simple differences in motion profiles between the tasks. To quantify the importance of a given coding dimension for task-decoding accuracy, we repeated the decoding procedure with individual dimensions (Fig. 6d).

### Software

For all data analysis (image preprocessing and population analysis) and statistics, we used the MATLAB programming environment (2016a).

### Statistics and reproducibility

For all analyses, including random sampling (for example, choice of trials used for subject alignment or decoding), we performed multiple repetitions with independently drawn samples. Based on these repetitions, we computed 95% bootstrapping CIs as follows: we sort all values and take the 3rd and 78th values as borders of the CI, as this interval contains 76 of 80 (that is, 95%) of the values. We then determine significance based on nonoverlapping CIs. If not indicated differently, violin plots indicate mean and 95% CIs. Box plots indicate median (center), 25th and 75th percentiles (box) and most extreme data points (whiskers) that were not considered outliers (points for which the distance from the box exceeds 1.5 times the length of the box). The sample sizes required for this study were initially estimated based on pilot behavior studies. No statistical method was used to predetermine sample size, but our sample sizes are similar to those reported in previous publications^8,41^. We excluded one animal because we did not observe any neuronal activity due to insufficient labeling and/or GRIN lens misplacement. In accordance with the animal welfare regulations, we had to terminate the behavior experiments for six mice because they did not learn the task sufficiently (performance below 50% after 3 days of training). We excluded eight imaging sessions (from a total of 132, 12 mice × 11 days) because we could not align the recorded frames to frames from previous sessions (Supplementary Video 4 and Extended Data Fig. 3b). The experiments were not randomized, and the investigators were not blinded to allocation during experiments and outcome assessment.

### Reporting summary

Further information on research design is available in the Nature Portfolio Reporting Summary linked to this article.

## Online content

Any methods, additional references, Nature Portfolio reporting summaries, source data, extended data, supplementary information, acknowledgements, peer review information; details of author contributions and competing interests; and statements of data and code availability are available at 10.1038/s41593-024-01704-5.

## Supplementary information

Reporting Summary

Supplementary Video 1 X and Y shuttling.

Supplementary Video 2 Simultaneous recordings of mouse behavior and neural activity.

Supplementary Video 3 Preprocessing of calcium imaging movies and the effect of lowpass filtering.

Supplementary Video 4 Alignment of imaging sessions.

## Source data

Source Data Extended Data Fig. 2a,c,d Unprocessed images.

## Data Availability

The source data that support the findings of this study are available at Zenodo at https://zenodo.org/records/11282437 (ref. ^42^). The raw imaging data will be made available upon reasonable request. Source data are provided with this paper.

## Code availability

The MATLAB codes detailing all aspects of the performed analysis are publicly available at Zenodo at https://zenodo.org/records/11283463 (ref. ^43^).

## References

[1] Le Merre, P., Ährlund-Richter, S. & Carlén, M. The mouse prefrontal cortex: unity in diversity. *Neuron* **109**, 1925–1944 (2021). 33894133 10.1016/j.neuron.2021.03.035

[2] Burgos-Robles, A., Vidal-Gonzalez, I. & Quirk, G. J. Sustained conditioned responses in prelimbic prefrontal neurons are correlated with fear expression and extinction failure. *J. Neurosci.* **29**, 8474–8482 (2009). 19571138 10.1523/JNEUROSCI.0378-09.2009 PMC2733220

[3] Otis, J. M. et al. Prefrontal cortex output circuits guide reward seeking through divergent cue encoding. *Nature* **543**, 103–107 (2017). 28225752 10.1038/nature21376 PMC5772935

[4] Courtin, J. et al. Prefrontal parvalbumin interneurons shape neuronal activity to drive fear expression. *Nature* **505**, 92–96 (2014). 24256726 10.1038/nature12755

[5] Karalis, N. et al. 4-Hz oscillations synchronize prefrontal-amygdala circuits during fear behavior. *Nat. Neurosci.* **19**, 605–612 (2016). 26878674 10.1038/nn.4251 PMC4843971

[6] Dejean, C. et al. Prefrontal neuronal assemblies temporally control fear behaviour. *Nature* **535**, 420–424 (2016). 27409809 10.1038/nature18630

[7] Diehl, M. M. et al. Divergent projections of the prelimbic cortex bidirectionally regulate active avoidance. *eLife* **9**, e59281 (2020). 33054975 10.7554/eLife.59281 PMC7588229

[8] Jercog, D. et al. Dynamical prefrontal population coding during defensive behaviours. *Nature* **595**, 690–694 (2021). 34262175 10.1038/s41586-021-03726-6

[9] Le Merre, P. et al. Reward-based learning drives rapid sensory signals in medial prefrontal cortex and dorsal hippocampus necessary for goal-directed behavior. *Neuron* **97**, 83–91 (2018). 29249287 10.1016/j.neuron.2017.11.031 PMC5766832

[10] Murugan, M. et al. Combined social and spatial coding in a descending projection from the prefrontal cortex. *Cell* **171**, 1663–1677 (2017). 29224779 10.1016/j.cell.2017.11.002 PMC5889923

[11] Burgos-Robles, A. et al. Amygdala inputs to prefrontal cortex guide behavior amid conflicting cues of reward and punishment. *Nat. Neurosci.* **20**, 824–835 (2017). 28436980 10.1038/nn.4553 PMC5448704

[12] Siniscalchi, M. J., Phoumthipphavong, V., Ali, F., Lozano, M. & Kwan, A. C. Fast and slow transitions in frontal ensemble activity during flexible sensorimotor behavior. *Nat. Neurosci.* **19**, 1234–1242 (2016). 27399844 10.1038/nn.4342 PMC5003707

[13] Schmitt, L. I. et al. Thalamic amplification of cortical connectivity sustains attentional control. *Nature* **545**, 219–223 (2017). 28467827 10.1038/nature22073 PMC5570520

[14] Huda, R. et al. Distinct prefrontal top–down circuits differentially modulate sensorimotor behavior. *Nat. Commun.* **11**, 6007 (2020). 33243980 10.1038/s41467-020-19772-z PMC7691329

[15] Stringer, C. et al. Spontaneous behaviors drive multidimensional, brainwide activity. *Science* **364**, 255 (2019). 31000656 10.1126/science.aav7893 PMC6525101

[16] Musall, S., Kaufman, M. T., Juavinett, A. L., Gluf, S. & Churchland, A. K. Single-trial neural dynamics are dominated by richly varied movements. *Nat. Neurosci.* **22**, 1677–1686 (2019). 31551604 10.1038/s41593-019-0502-4 PMC6768091

[17] Steinmetz, N. A., Zatka-Haas, P., Carandini, M. & Harris, K. D. Distributed coding of choice, action and engagement across the mouse brain. *Nature* **576**, 266–273 (2019). 31776518 10.1038/s41586-019-1787-x PMC6913580

[18] Rigotti, M. et al. The importance of mixed selectivity in complex cognitive tasks. *Nature* **497**, 585–590 (2013). 23685452 10.1038/nature12160 PMC4412347

[19] Galgali, A. R., Sahani, M. & Mante, V. Residual dynamics resolves recurrent contributions to neural computation. *Nat. Neurosci.* **26**, 326–338 (2023). 36635498 10.1038/s41593-022-01230-2

[20] Safaie, M. et al. Preserved neural population dynamics across animals performing similar behaviour. *Nature* **623**, 765–771 (2023). 37938772 10.1038/s41586-023-06714-0 PMC10665198

[21] Mante, V., Sussillo, D., Shenoy, K. V. & Newsome, W. T. Context-dependent computation by recurrent dynamics in prefrontal cortex. *Nature* **503**, 78–84 (2013). 24201281 10.1038/nature12742 PMC4121670

[22] Peters, A. J., Fabre, J. M. J., Steinmetz, N. A., Harris, K. D. & Carandini, M. Striatal activity topographically reflects cortical activity. *Nature* **591**, 420–425 (2021). 33473213 10.1038/s41586-020-03166-8 PMC7612253

[23] Kyriazi, P., Headley, D. B. & Paré, D. Different multidimensional representations across the amygdalo-prefrontal network during an approach-avoidance task. *Neuron* **107**, 717–730 (2020). 32562662 10.1016/j.neuron.2020.05.039 PMC7442738

[24] Fusi, S., Miller, E. K. & Rigotti, M. Why neurons mix: high dimensionality for higher cognition. *Curr. Opin. Neurobiol.* **37**, 66–74 (2016). 26851755 10.1016/j.conb.2016.01.010

[25] Vyas, S., Golub, M. D., Sussillo, D. & Shenoy, K. V. Computation through neural population dynamics. *Annu. Rev. Neurosci.* **43**, 249–275 (2020). 32640928 10.1146/annurev-neuro-092619-094115 PMC7402639

[26] Diehl, M. M. et al. Active avoidance requires inhibitory signaling in the rodent prelimbic prefrontal cortex. *eLife* **7**, e34657 (2018). 29851381 10.7554/eLife.34657 PMC5980229

[27] Moscarello, J. M. & LeDoux, J. E. Active avoidance learning requires prefrontal suppression of amygdala-mediated defensive reactions. *J. Neurosci.* **33**, 3815–3823 (2013). 23447593 10.1523/JNEUROSCI.2596-12.2013 PMC3607300

[28] Kajs, B. L., Loewke, A. C., Dorsch, J. M., Vinson, L. T. & Gunaydin, L. A. Divergent encoding of active avoidance behavior in corticostriatal and corticolimbic projections. *Sci. Rep.* **12**, 10731 (2022). 35750718 10.1038/s41598-022-14930-3 PMC9232563

[29] Vander Weele, C. M. et al. Dopamine enhances signal-to-noise ratio in cortical-brainstem encoding of aversive stimuli. *Nature* **563**, 397–401 (2018). 30405240 10.1038/s41586-018-0682-1 PMC6645392

[30] Christensen, A. J., Ott, T. & Kepecs, A. Cognition and the single neuron: how cell types construct the dynamic computations of frontal cortex. *Curr. Opin. Neurobiol.* **77**, 102630 (2022). 36209695 10.1016/j.conb.2022.102630 PMC10375540

[31] Pinto, L. & Dan, Y. Cell-type-specific activity in prefrontal cortex during goal-directed behavior. *Neuron* **87**, 437–450 (2015). 26143660 10.1016/j.neuron.2015.06.021 PMC4506259

[32] Schneider, D. M., Sundararajan, J. & Mooney, R. A cortical filter that learns to suppress the acoustic consequences of movement. *Nature* **561**, 391–395 (2018). 30209396 10.1038/s41586-018-0520-5 PMC6203933

[33] Choi, J.-S., Cain, C. K. & LeDoux, J. E. The role of amygdala nuclei in the expression of auditory signaled two-way active avoidance in rats. *Learn. Mem.* **17**, 139–147 (2010). 20189958 10.1101/lm.1676610 PMC2832923

[34] Ramirez, F., Moscarello, J. M., LeDoux, J. E. & Sears, R. M. Active avoidance requires a serial basal amygdala to nucleus accumbens shell circuit. *J. Neurosci.* **35**, 3470–3477 (2015). 25716846 10.1523/JNEUROSCI.1331-14.2015 PMC4339356

[35] Malagon-Vina, H., Ciocchi, S., Passecker, J., Dorffner, G. & Klausberger, T. Fluid network dynamics in the prefrontal cortex during multiple strategy switching. *Nat. Commun.* **9**, 309 (2018). 29358717 10.1038/s41467-017-02764-x PMC5778086

[36] Rikhye, R. V., Gilra, A. & Halassa, M. M. Thalamic regulation of switching between cortical representations enables cognitive flexibility. *Nat. Neurosci.* **21**, 1753–1763 (2018). 30455456 10.1038/s41593-018-0269-z PMC7225728

[37] Reinert, S., Hübener, M., Bonhoeffer, T. & Goltstein, P. M. Mouse prefrontal cortex represents learned rules for categorization. *Nature* **593**, 411–417 (2021). 33883745 10.1038/s41586-021-03452-z PMC8131197

[38] Thévenaz, P., Ruttimann, U. E. & Unser, M. A pyramid approach to subpixel registration based on intensity. *IEEE Trans. Image Process.* **7**, 27–41 (1998). 18267377 10.1109/83.650848

[39] Mukamel, E. A., Nimmerjahn, A. & Schnitzer, M. J. Automated analysis of cellular signals from large-scale calcium imaging data. *Neuron* **63**, 747–760 (2009). 19778505 10.1016/j.neuron.2009.08.009 PMC3282191

[40] Mathis, A. et al. DeepLabCut: markerless pose estimation of user-defined body parts with deep learning. *Nat. Neurosci.* **21**, 1281–1289 (2018). 30127430 10.1038/s41593-018-0209-y

[41] Grewe, B. F. et al. Neural ensemble dynamics underlying a long-term associative memory. *Nature* **543**, 670–675 (2017). 28329757 10.1038/nature21682 PMC5378308

[42] Ehret, B. Data associated with the publication ‘Population-level coding of avoidance learning in medial prefrontal cortex’ by Benjamin Ehret et al. *Zenodo* https://zenodo.org/records/11282437 (2024). 10.1038/s41593-024-01704-5 PMC11374698 39075325

[43] Ehret, B. behret/paper_code_active_avoidance: analysis_code_v1. *Zenodo* https://zenodo.org/records/11283463 (2024).

[44] Paxinos, G. & Franklin, K. B. J. *The Mouse Brain in Stereotaxic Coordinates* (Gulf Professional Publishing, 2004).

