# Supplementary material for: Population-level coding of avoidance learning in medial prefrontal cortex
Source: Nat Neurosci. 2024 Jul 29;27(9):1805–15. doi: 10.1038/s41593-024-01704-5 (PMC11374698; doi:10.1038/s41593-024-01704-5)
Supplement: Supplementary file 1 — Reporting Summary [file 41593_2024_1704_MOESM1_ESM.pdf]

nature portfolio

Corresponding author(s): Benjamin Ehret, Benjamin F. Grewe

Last updated by author(s): May 24, 2024

Reporting Summary

Nature Portfolio wishes to improve the reproducibility of the work that we publish. This form provides structure for consistency and transparency in reporting. For further information on Nature Portfolio policies, see our Editorial Policies and the Editorial Policy Checklist.

Statistics

For all statistical analyses, confirm that the following items are present in the figure legend, table legend, main text, or Methods section.

n/a	Confirmed	
☐	☒	The exact sample size (*n*) for each experimental group/condition, given as a discrete number and unit of measurement
☐	☒	A statement on whether measurements were taken from distinct samples or whether the same sample was measured repeatedly
☐	☒	The statistical test(s) used AND whether they are one- or two-sided<br>*Only common tests should be described solely by name; describe more complex techniques in the Methods section.*
☒	☐	A description of all covariates tested
☒	☐	A description of any assumptions or corrections, such as tests of normality and adjustment for multiple comparisons
☐	☒	A full description of the statistical parameters including central tendency (e.g. means) or other basic estimates (e.g. regression coefficient) AND variation (e.g. standard deviation) or associated estimates of uncertainty (e.g. confidence intervals)
☐	☒	For null hypothesis testing, the test statistic (e.g. *F*, *t*, *r*) with confidence intervals, effect sizes, degrees of freedom and *P* value noted<br>*Give P values as exact values whenever suitable.*
☒	☐	For Bayesian analysis, information on the choice of priors and Markov chain Monte Carlo settings
☒	☐	For hierarchical and complex designs, identification of the appropriate level for tests and full reporting of outcomes
☐	☒	Estimates of effect sizes (e.g. Cohen's *d*, Pearson's *r*), indicating how they were calculated

Our web collection on statistics for biologists contains articles on many of the points above.

Software and code

Policy information about availability of computer code

Data collection	All in vivo calcium imaging data was collected with the Inscopix miniaturized microscope system nVista 2.0.<br>All histological images were acquired using a either CLSM - Leica Stellaris 5 upright microscope equipped with an HC PL APO CS2 20x 0.75 IMM 0.66 objective (data acquisition software used was LAS X) or an Olympus fluorescence microscope (BX51).<br>All behavior videos were acquired using two top view B/W cameras (DMK 23FV024; ImagingSource) and custom written MATLAB code (2016a).
Data analysis	For all imaging data analysis we used the MATLAB programming environment (2016a) and developed custom code for (1) the imaging data preprocessing, (2) for the cell extraction, (3) for the joint subspace alignment across all mice and (4) for the population data analysis. For motion correction of the calcium imaging movies we used the Turboreg software. For tracking of the animals we used the DeepLabCut software. All code required to reproduce the findings of the paper is available at https://zenodo.org/records/11283463.

For manuscripts utilizing custom algorithms or software that are central to the research but not yet described in published literature, software must be made available to editors and reviewers. We strongly encourage code deposition in a community repository (e.g. GitHub). See the Nature Portfolio guidelines for submitting code & software for further information.

Data

Policy information about availability of data

All manuscripts must include a data availability statement. This statement should provide the following information, where applicable:

- Accession codes, unique identifiers, or web links for publicly available datasets
- A description of any restrictions on data availability
- For clinical datasets or third party data, please ensure that the statement adheres to our policy

The source data that support the findings of this study are available at https://zenodo.org/records/11282437. The raw imaging data will be made available upon reasonable request.

Research involving human participants, their data, or biological material

Policy information about studies with human participants or human data. See also policy information about sex, gender (identity/presentation), and sexual orientation and race, ethnicity and racism.

Reporting on sex and gender	N/A
Reporting on race, ethnicity, or other socially relevant groupings	N/A
Population characteristics	N/A
Recruitment	N/A
Ethics oversight	N/A

Note that full information on the approval of the study protocol must also be provided in the manuscript.

Field-specific reporting

Please select the one below that is the best fit for your research. If you are not sure, read the appropriate sections before making your selection.

☒ Life sciences ☐ Behavioural & social sciences ☐ Ecological, evolutionary & environmental sciences

For a reference copy of the document with all sections, see nature.com/documents/nr-reporting-summary-flat.pdf

Life sciences study design

All studies must disclose on these points even when the disclosure is negative.

Sample size	Sample sizes were selected based on previous experience from related research and literature:<br>- Hippocampus and cognitive control group size n = 8 - 12 (Chung et al., 2021)<br>- ACC and avoidance behaviour group size n = 7 - 14 (Lee et al., 2019)<br>- Amygdala and stated dependent flexibility group size n = 10 (Fustiñana et al., 2021)<br>- Neural ensemble dynamics in the amygdala group size n = 8-12 (paper from study director: Grewe et al., 2017)
Data exclusions	In accordance with the animal welfare regulations, we had to terminate the behavior experiments for 6 mice because they did not learn the task sufficiently (performance below 50% after 3 days of training). We excluded 8 imaging sessions (from a total of 132, 12 mice x 11 days) because we could not align the recorded frames to frames from previous sessions (see Supplementary Movie 4, Fig. S3B).
Replication	Results that are displayed using representative examples and were not statistically evaluated (Extended Data Fig. 2C-D and Extended Data Fig. 3A) were repeated multiple times and we ensured that all repetitions produced qualitatively similar results.
Randomization	We did not randomize as we only had one group of animals that we recorded from.
Blinding	B.E. alone performed all animal experiments. Since animals need to be prepared and handled individually and due to the nature of the experiment a blinding of the experimenter was not possible.

Reporting for specific materials, systems and methods

We require information from authors about some types of materials, experimental systems and methods used in many studies. Here, indicate whether each material, system or method listed is relevant to your study. If you are not sure if a list item applies to your research, read the appropriate section before selecting a response.

Materials & experimental systems

n/a	Involved in the study	
☐	☒	Antibodies
☒	☐	Eukaryotic cell lines
☒	☐	Palaeontology and archaeology
☐	☒	Animals and other organisms
☒	☐	Clinical data
☒	☐	Dual use research of concern
☒	☐	Plants

Methods

n/a	Involved in the study	
☒	☐	ChIP-seq
☒	☐	Flow cytometry
☒	☐	MRI-based neuroimaging

Antibodies

Antibodies used

rabbit anti-GAD65 (1:500, AB1511, Millipore)
rabbit anti-Neurogranin (1:2000, 07-425, Millipore)
Alexa 594 anti-rabbit (1:200, A-11062, Invitorgen)
DAPI (1:1000, D1306, Invitrogen)
Nissel stain (NeuroTrace 530/615, N21482, Invitrogen)

Validation

Antibodies were chosen based on a literature review for each antibody to identify the best candidate for our experiments. Validation was determined by reviewing the manufacturer's literature, other published research, and prior experiments in the lab. For each experiment, at least one slide was designated for a "secondary only" control and examined for potential background staining.
Commercial antibodies were validated by the manufacturer:
rabbit anti-GAD65 www.merckmillipore.com/CH/de/product/Anti-Glutamate-Decarboxylase-65-67-Antibody,MM_NF-AB1511
rabbit anti-Neurogranin https://www.merckmillipore.com/CH/de/product/Anti-Neurogranin,MM_NF-07-425-I-100UG
Alexa 594 anti-rabbit www.thermofisher.com/antibody/product/Rabbit-anti-Mouse-IgG-H-L-Cross-Adsorbed-Secondary-Antibody-Polyclonal/A-11062
DAPI www.thermofisher.com/order/catalog/product/D1306
Nissel stain www.thermofisher.com/order/catalog/product/N21482

Animals and other research organisms

Policy information about studies involving animals; ARRIVE guidelines recommended for reporting animal research, and Sex and Gender in Research

Laboratory animals

Experiments were performed on adult male C57BL/6J Crl1. Mice were 10-17 weeks old at the time of the virus injection surgery and 20–32 weeks old during in vivo imaging and behavioral experiments. Animals were housed in individually ventilated cages (IVC) in a 12 h light/dark cycle room (lights on from 7:00 to 19:00, ambient temperature: 21-24°C, humidity: 35-70%), and were provided food and water ad libitum. After import from the breeders, mice were given a 2 weeks acclimatization period to the new housing condition prior to the first surgery. During the experiments mice were kept in groups of 2 to 5 animals.

Wild animals

No wild animals were used in the study.

Reporting on sex

We report the sex of all animals used for this study in the methods section (all male).

Field-collected samples

No field collected samples were used in the study.

Ethics oversight

All animal well-fare and ethical aspects of our study were evaluated by the Swiss Cantonal Veterinary office and approved,.

Note that full information on the approval of the study protocol must also be provided in the manuscript.
