# Supplementary figures and images for: Population-level coding of avoidance learning in medial prefrontal cortex
Source: Nat Neurosci. 2024 Jul 29;27(9):1805–15. doi: 10.1038/s41593-024-01704-5 (PMC11374698; doi:10.1038/s41593-024-01704-5)

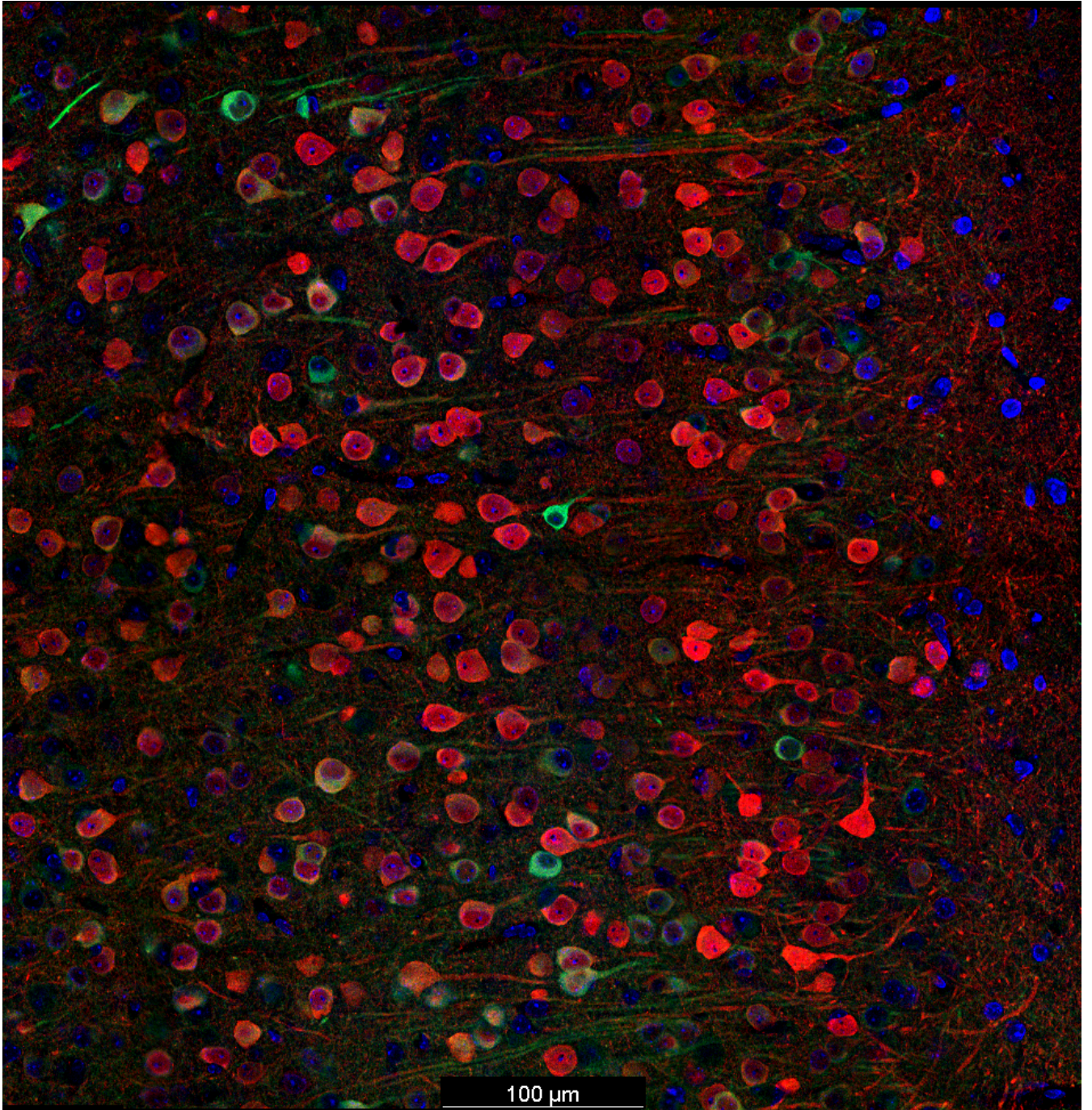

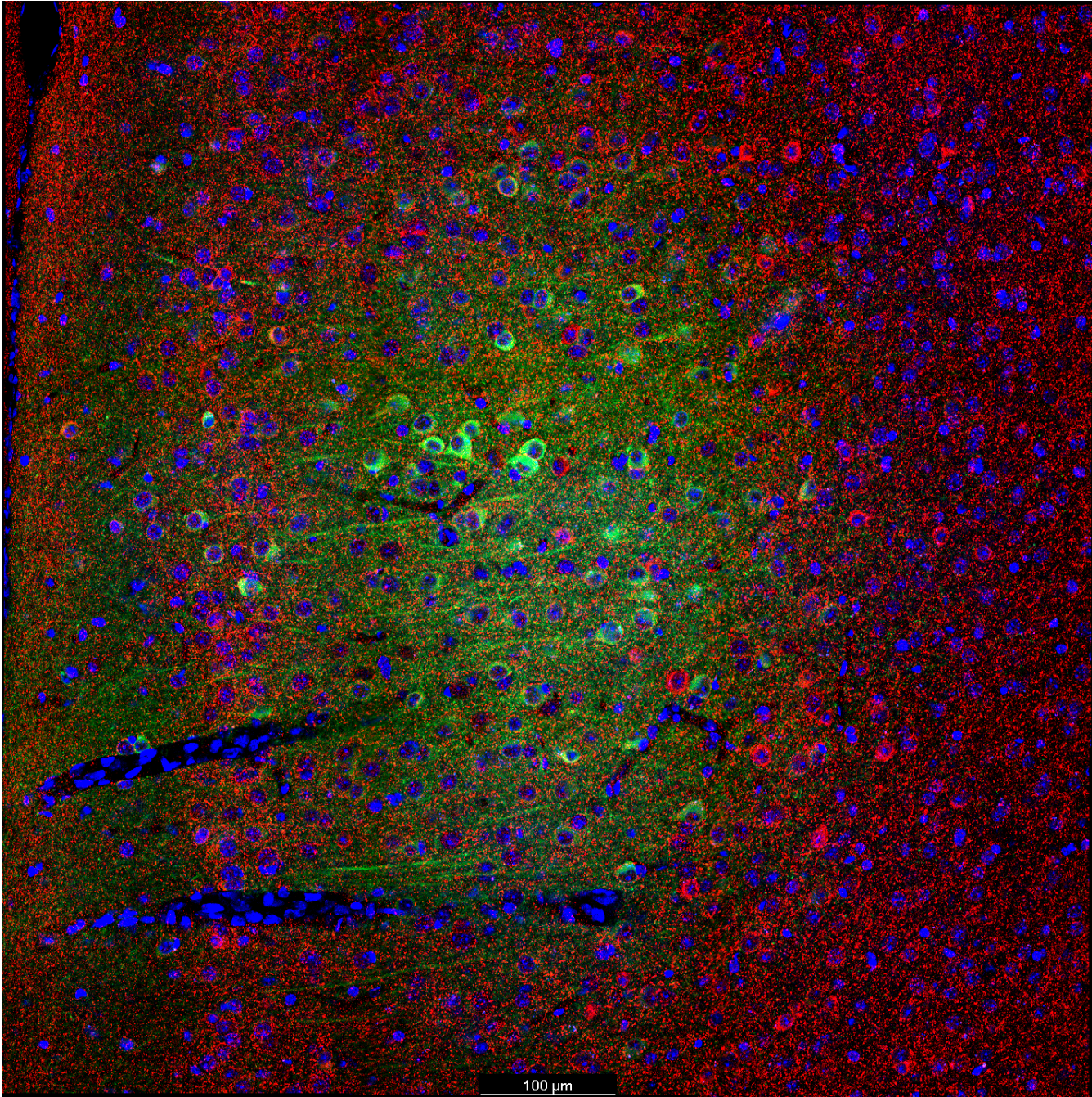

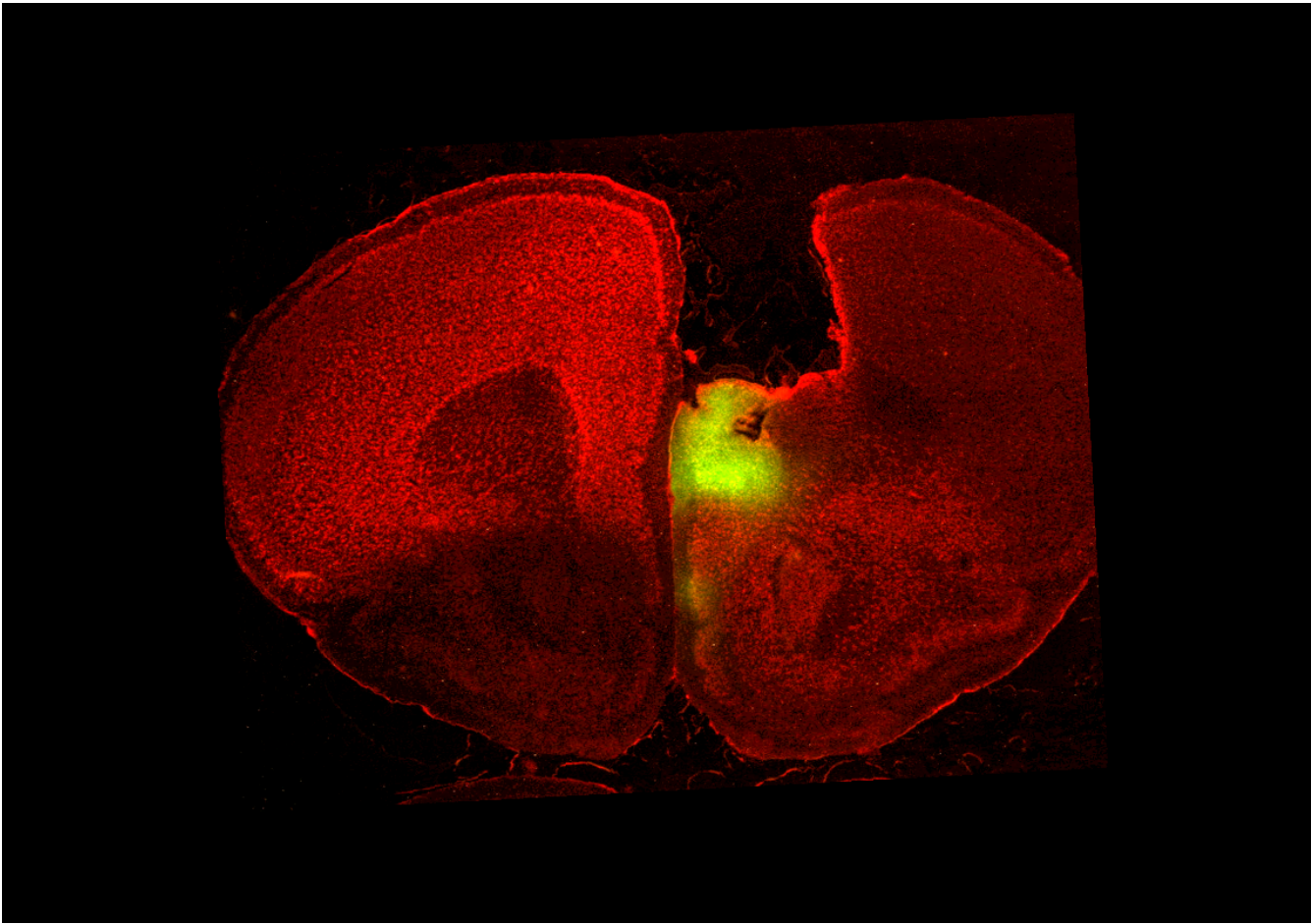

Supplement: Supplementary file 6 — Unprocessed images. [file 41593_2024_1704_MOESM6_ESM.pdf]
